# Unraveling the metabolic heterogeneity and commonality in senescent cells using systems modeling

**DOI:** 10.1093/lifemedi/lnaf003

**Published:** 2025-01-20

**Authors:** Gong-Hua Li, Yu-Hong Li, Qin Yu, Qing-Qing Zhou, Run-Feng Zhang, Chong-Jun Weng, Ming-Xia Ge, Qing-Peng Kong

**Affiliations:** State Key Laboratory of Genetic Evolution & Animal Models, Key Laboratory of Healthy Aging Research of Yunnan Province, Kunming Key Laboratory of Healthy Aging Study, Kunming Institute of Zoology, Chinese Academy of Sciences, Kunming 650201, China; College of Biological Resources and Food Engineering, Qujing Normal University, Qujing 655000, China; State Key Laboratory of Genetic Evolution & Animal Models, Key Laboratory of Healthy Aging Research of Yunnan Province, Kunming Key Laboratory of Healthy Aging Study, Kunming Institute of Zoology, Chinese Academy of Sciences, Kunming 650201, China; State Key Laboratory of Genetic Evolution & Animal Models, Key Laboratory of Healthy Aging Research of Yunnan Province, Kunming Key Laboratory of Healthy Aging Study, Kunming Institute of Zoology, Chinese Academy of Sciences, Kunming 650201, China; State Key Laboratory of Genetic Evolution & Animal Models, Key Laboratory of Healthy Aging Research of Yunnan Province, Kunming Key Laboratory of Healthy Aging Study, Kunming Institute of Zoology, Chinese Academy of Sciences, Kunming 650201, China; State Key Laboratory of Genetic Evolution & Animal Models, Key Laboratory of Healthy Aging Research of Yunnan Province, Kunming Key Laboratory of Healthy Aging Study, Kunming Institute of Zoology, Chinese Academy of Sciences, Kunming 650201, China; State Key Laboratory of Genetic Evolution & Animal Models, Key Laboratory of Healthy Aging Research of Yunnan Province, Kunming Key Laboratory of Healthy Aging Study, Kunming Institute of Zoology, Chinese Academy of Sciences, Kunming 650201, China; State Key Laboratory of Genetic Evolution & Animal Models, Key Laboratory of Healthy Aging Research of Yunnan Province, Kunming Key Laboratory of Healthy Aging Study, Kunming Institute of Zoology, Chinese Academy of Sciences, Kunming 650201, China; CAS Center for Excellence in Animal Evolution and Genetics, Chinese Academy of Sciences, Kunming 650223, China

**Keywords:** cellular senescence, metabolic profile, metabolic modeling, meta-analysis

## Abstract

Cellular senescence is a key contributor to aging and aging-related diseases, but its metabolic profiles are not well understood. Here, we performed a systematic analysis of the metabolic features of four types of cellular senescence (replication, irradiation, reactive oxygen species [ROS], and oncogene) in 12 cell lines using genome-wide metabolic modeling and meta-analysis. We discovered that replicative and ROS-induced senescence share a common metabolic signature, marked by decreased lipid metabolism and downregulated mevalonate pathway, while irradiation and oncogene-induced senescence exhibit more heterogeneity and divergence. Our genome-wide knockout simulations showed that enhancing the mevalonate pathway, by administrating mevalonate for instance, could reverse the metabolic alterations associated with senescence and human tissue aging, suggesting a potential anti-aging or lifespan-extending effect. Indeed, the experiment in *Caenorhabditis elegans* showed that administrating mevalonate significantly increased the lifespan. Our study provides a new insight into the metabolic landscape of cell senescence and identifies potential targets for anti-aging interventions.

## Introduction

Cellular senescence is a state of cell cycle arrest in which proliferating cells become resistant to growth-promoting stimuli but remain viable and metabolically active after prolonged time [1]. Cell senescence has some beneficial roles in embryonic development [2], wound healing [3], and tumor suppression [4], but it also has deleterious effects on human health: it plays a pivotal role in aging and age-related diseases [5], such as cancer [6], cardiovascular disease [7], diabetes [8], and neurodegeneration [9]. Senescent cells accumulate in tissues during aging and secrete a variety of pro-inflammatory and pro-fibrotic factors, collectively known as the senescence-associated secretory phenotype (SASP) that can impair tissue function and promote aging and age-related diseases [10]. Even a small fraction of senescent cells can induce aging phenotypes in young animal models [8]. Therefore, modulating senescence may offer novel therapeutic strategies for enhancing health span and life span [11]. For example, genetic or pharmacological removal of senescent cells has been shown to extend life span and improve health span in animal models [12].

The metabolic activity of senescent cells is important for supporting their survival and function, as well as for modulating their interactions with the surrounding microenvironment [13]. For instance, senescent cells can alter the nutrient availability, oxygen consumption, and extracellular matrix composition of the tissue niche, thereby influencing the behavior and fate of neighboring cells [14]. Some studies have reported that senescent cells show reduced mitochondrial function and increased ROS production, which cause protein and lipid damage [15, 16]. However, the metabolic consequences of cellular senescence are still poorly understood and elusive.

There are different types of cellular senescence that have been identified, including oncogene-induced senescence (OIS), irradiation-induced senescence (IIS), stress-induced premature senescence (SIPS, e.g. ROS-induced senescence), and replicative senescence (RS) [17]. Different senescent types have distinct pathological and physiological significance including chronic diseases and aging-related disorders [1, 18]. For instance, OIS serves as a safeguard against cancer by arresting the proliferation of potentially malignant cells [19]. IIS aids in tissue repair post-injury but may lead to fibrosis if unresolved [20]. Stress-induced senescence, exemplified by ROS-induced senescence, offers protection against damage but can result in chronic inflammation and tissue dysfunction if senescent cells accumulate [21]. RS signifies the natural cellular aging process, contributing to the decline in tissue function and regenerative ability [22]. Although these different types of senescence share some common features, such as cell cycle arrest, DNA damage response activation, cyclin-dependent kinase inhibitor expression, p53 stabilization, and SASP induction [23], they also have distinct characteristics, such as the type and extent of DNA damage, the involvement of different signaling pathways, the expression of specific markers, and the duration and reversibility of the senescent state [24]. We asked whether these different types of senescent cells have common metabolic profiles or not and whether we can identify common key metabolic pathways and genes involved in senescence that may have potential to be intervene targets.

In this study, we performed genome-wide metabolic modeling and meta-analysis of 178 cell samples from 27 studies, covering 12 cell lines and 4 cellular senescence types. We found that replication and ROS-induced senescence share common significant metabolic changes, such as decreased fatty acid metabolism, purine/pyrimidine synthesis defects, and impaired mevalonate-related pathways. We then used all-against-all knock-out analysis algorithm [25] to systematically identify the key genes and endogenous metabolites that may causally affect the senescence-related metabolic changes. Our study provides a systemic view of metabolic changes during cell senescence and identifies key genes and metabolites as potential nutrition/drugs for cellular aging prevention.

## Results

### Study design of genome-wide precision metabolic modeling of cellular senescence

**Figure 1.**
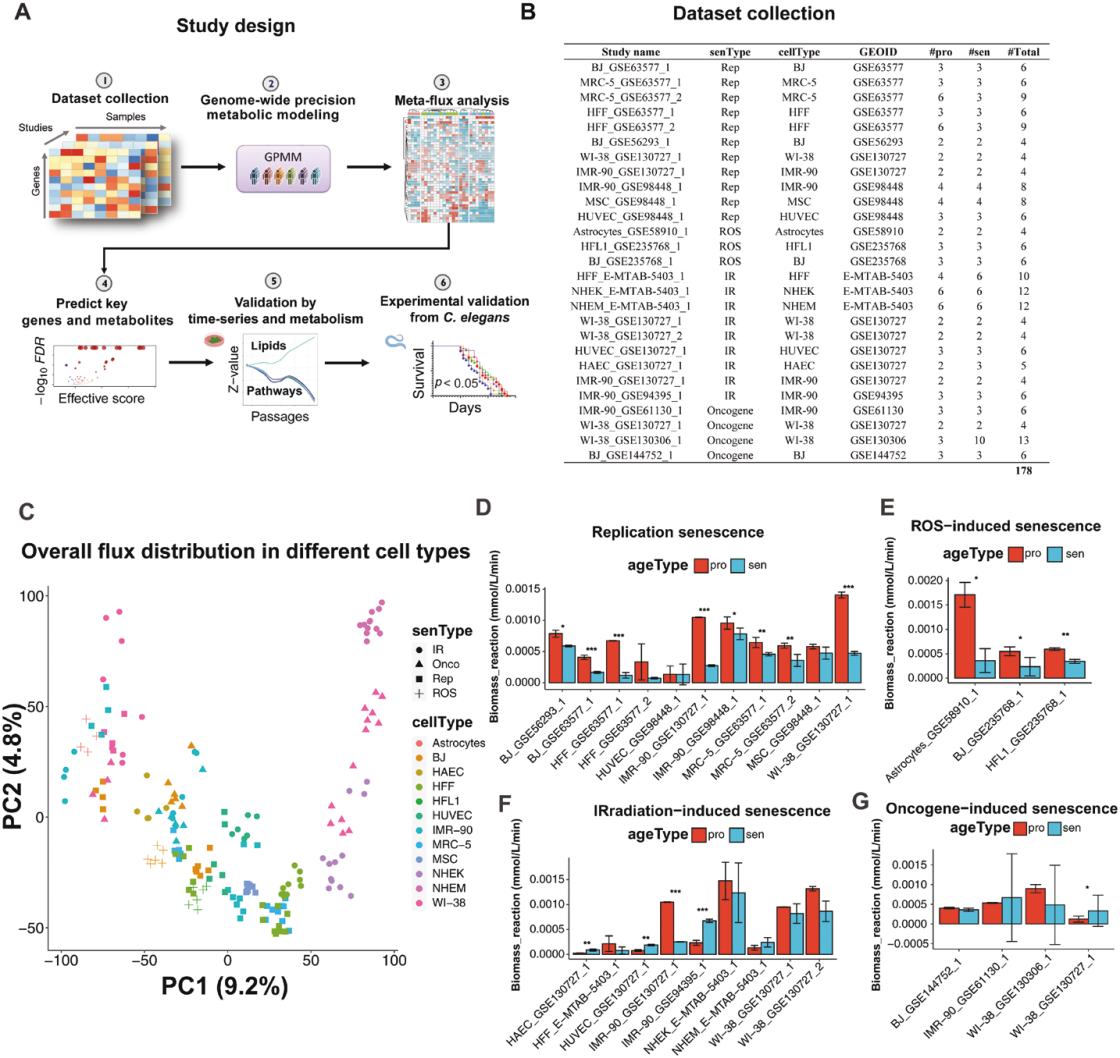
**Study design and data overview.** (A) Flowchart of study design. RNA-seq data was retrieved from public databases (NCBI and EBI). Genome-wide precision metabolic modeling was performed for each study using GPMM method. Meta-flux analysis was then performed to obtain metabolic profiles in different types of cellular senescence. Key genes and metabolites that modulate metabolic changes were predicted. The findings were validated using time-series analysis and metabolism data. Finally, the prediction was experimentally validated using the small animal model (*C. elegans*). (B) List of datasets for metabolic modeling and meta-analysis. (C) Principal component analysis (PCA) analysis of flux distribution in different cellular senescence types. “IR”, “Onco”, “Rep”, and “ROS” stand for IIS, OIS, RS, and ROIS, respectively. (D–G) Comparison between biomass reaction in proliferative and senescence samples in different types of cellular senescence. The *p*-values of < 0.05, < 0.01, and < 0.001 are represented by *, **, and ***, respectively. “pro” represents proliferative and “sen” represents senescence.

To elucidate the metabolic profiles of different types of senescent cells, we used genome-wide precision metabolic modeling (GPMM) [26] and compared them with those of proliferative cells in various cellular senescence types (Fig. 1A). We first retrieved RNA-seq data from public databases (NCBI and EBI) and collected 178 cell samples from 27 cellular senescence studies, covering 12 cell lines and four senescence types: 11 RS studies, 3 ROS-induced senescence (ROIS) studies, 9 IIS studies, and 4 OIS studies (Fig. 1B). Second, we used our recently developed GPMM method [26] to generate the metabolic profiles for each study and performed flux meta-analysis for each senescence type to identify the metabolic changes across multiple studies. Third, we conducted an all-against-all metabolic knockout analysis using our recently developed methods (FastMM) [25] to discover the potential key genes and key metabolites that causally affect the altered metabolic profiles in senescent cells. Fourthly, to complement the meta-analysis based on case–control studies, we further examined the dynamic changes from proliferation to senescence and tested the consistency of the findings from the meta-analysis in time-series analysis. In addition, we conducted LC/MS-based metabolomics to validate the common metabolic changes among meta-analysis and time-series analysis. Finally, we experimentally validated the predicted key metabolite and explored the possible underlying mechanisms in *Caenorhabditis elegans* (*C. elegans*).

### Common and heterogeneous flux changes among different types of cellular senescence

We used GPMM to construct metabolic networks for the 178 cell samples, containing 2963 reactions, 1463 metabolites and 2248 genes. The fluxes among these samples clustered by cell type (Fig. 1C). The “biomass reaction,” a surrogate measure for cell proliferation or growth, integrates essential cellular components, such as DNA, RNA, proteins, and lipids. A greater magnitude of the biomass reaction is indicative of an elevated cell growth rate. We found that the predicted biomass reactions were consistently and significantly lower in senescent cells than in proliferative cells in RS (8/11, 73%, *P* < 0.05) and ROIS (3/3, 100%, *P *< 0.05), indicating that we successfully captured the growth arrest phenomena in these two types of senescence (Fig. 1D and 1E). For IIS and OIS, we did not observe a common pattern of growth arrest (Fig. 1F and 1G).

**Figure 2.**
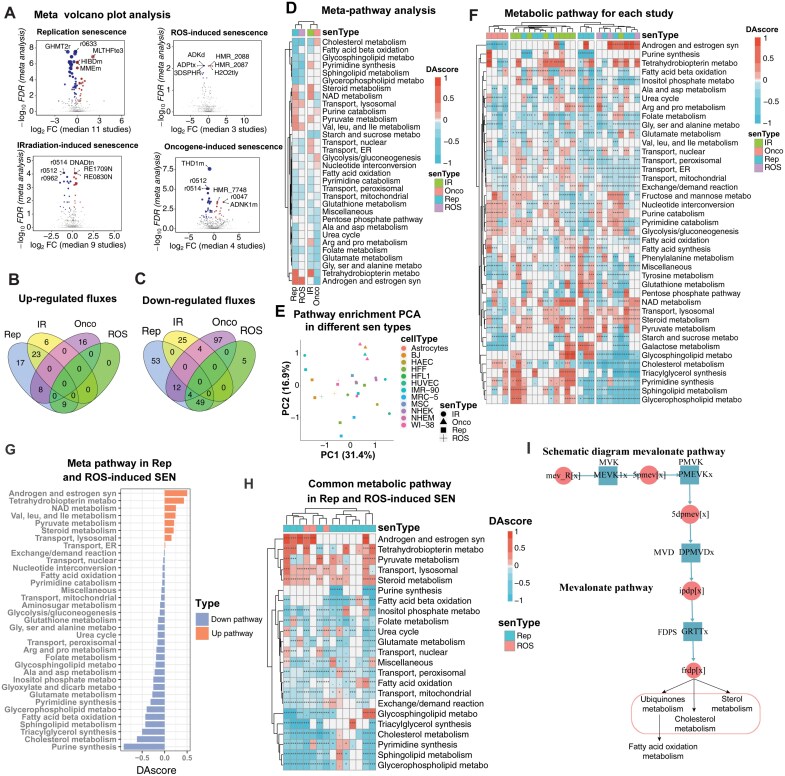
**Meta-flux analysis in different types of cellular senescence.** (A) Meta volcano plot for four different types of senescence (replication, ROS-induced, irradiation-induced, and oncogene-induced). *X*-axis represents the mean log_2_(fold change (FC)) among studies in a given senescent type, while *Y*-axis represents the −log_10_ (false discovery rate (FDR)). Reactions with fluxes of FDR < 0.05 and mean log_2_FC > 0 are colored red, and reactions with fluxes of FDR < 0.05 and mean log_2_FC < 0 are colored blue. (B, C) Overlapped up-regulated and down-regulated fluxes in different types of cellular senescence. (D) Meta-pathway analysis in these four senescence types. Pathway enrichment was measured by DAscore (range from −1 to 1), where positive and negative values indicate increased and decreased metabolic pathways, respectively. (E) PCA analysis of DAscore for each study. The plot was performed based on the PC1 and PC2. (F) Heatmap of metabolic pathway changes for each study. Pathway enrichment was also measured by DAscore. The FDR of < 0.05, < 0.01, and < 0.001 are represented by *, **, and ***, respectively. (G) Meta-pathway analysis by combining replication and ROS-induced cellular senescence. “Rep” denotes replication, while “SEN” refers to senescence. “syn” and “metabo” are abbreviations for synthesis and metabolism, respectively. (H) Heatmap of common metabolic pathways in replication and ROS-induced cellular senescence. (I) Schematic diagram mevalonate pathway, related to Fig. S1.

We then performed flux meta-analysis for each senescence type across multiple studies (Tables S1–S4), and identified 57, 9, 29, and 24 up-regulated fluxes in RS, ROIS, IIS and OIS senescent types, respectively (FDR < 0.05, Fig. 2A and 2B). Notably, all of the up-regulated fluxes in ROIS were also up-regulated in RS (9/9, 100%, Fig. 2B). In addition, we also identified 118, 58, 29, and 117 down-regulated fluxes in RS, ROIS, IIS and OIS senescent types, respectively (FDR < 0.05, Fig. 2A and 2C). Similarly, 53 of 58 (91%) down-regulated fluxes in ROIS were also down-regulated in RS (Fig. 2C). These results showed that ROIS and RS shared common metabolic changes.

For IIS or OIS, they differed from other senescence types. For example, in oncogene-induced senescence, only 8/24, 0/24, 0/24 of up-regulated fluxes in OIS overlapped with up-regulated fluxes in RS, ROIS, and IIS, respectively. Similarly, only 16/117, 4/117, 4/117 of down-regulated fluxes in OIS overlapped with down-regulated fluxes in RS, ROIS, and IIS (Fig. 2C). For IIS, although 23/29 up-regulated fluxes overlapped with RS, none of them overlapped with ROIS or OIS senescence (Fig. 2B). Similarly, only 0/29, 0/29, and 4/29 down-regulated fluxes overlapped with RS, ROIS and OIS senescence, respectively (Fig. 2C).

### Common and heterogeneous metabolic pathway alteration among different types of cellular senescence

We next investigated which pathways are altered in different types of cellular senescence by using meta-analysis of flux pathway analysis. We first performed the differential pathway analysis to find the significantly changed metabolic pathways using the meta-analysis-based fluxes changes for each type of cellular senescence using the DAscore method [26, 27]. The result showed that 32 metabolic pathways changed in at least two senescence types, and RS and ROS-induced cellular senescence clustered together (Fig. 2D). We then examined the significant pathways for each study and performed the principal component analysis (PCA), and also observed that ROS and replication-induced cell senescence clustered together and the IIS were scattered, while the OIS were distinct from the other three types (Fig. 2E). In addition, we noted that the changes in pathways appeared to be independent of cell type (Fig. 2E). For instance, both RS and ROIS formed clusters regardless of the cell types involved, and similarly, oncogene-induced senescence clustered cohesively across three different cell types, as depicted in Fig. 2E. These findings suggested that different cell types exhibit shared metabolic alterations within a specific type of replication/ROS or oncogene-induced cellular senescence.

To further determine the common and/or heterogeneous metabolic pathway changes in different cellular senescence, we compared the studies clustered based on the DAscore (Fig. 2F). Consistent with the meta-analysis and PCA analysis (Fig. 2D and 2E), the result showed that ROS and replication-induced cell senescence clustered together and had the greatest number of consistent metabolic pathway changes (Fig. 2F). Interestingly, we observed that IIS had the lowest consistency of metabolic pathways and the cluster was messy. Also consistent with the meta-analysis and PCA analysis, we also observed that OIS was distinct from the other three types (Fig. 2F). Together, these results showed the common fluxes and metabolic pathways in RS and ROIS senescence but heterogeneity in IR and OIS.

Therefore, we next focused on the common metabolic profiles in ROIS and RS cell senescence. The results revealed that the most significant common altered pathways in these two senescence types were the down-regulated purine and pyrimidine synthesis, lipid metabolism (including fatty acid oxidation, fatty acid beta oxidation, triacylglycerol synthesis, cholesterol metabolism, sphingolipid metabolism, glycerophospholipid metabolism, and glycosphingolipid metabolism), and the lipid metabolism related transports (peroxisomal transport and mitochondrial transport) (FDR < 0.05, Fig. 2G). These results were further confirmed by individual studies (Fig. 2H). For example, among the 14 studies with significant changes in lipid-related pathways, 89% (8/9), 89% (8/9), 86% (6/7), 100% (10/10), 91% (10/11), 100% (11/11), 78% (7/9), 91% (10/11), and 90% (9/10) studies showed down-regulation in fatty acid oxidation, fatty acid beta oxidation, triacylglycerol synthesis, cholesterol metabolism, sphingolipid metabolism, glycerophospholipid metabolism, glycosphingolipid metabolism, peroxisomal transport, and mitochondrial transport, respectively (Fig. 2H). Consistently, we observed that 28 of 29 significantly changed core lipid reactions were down-regulated from meta-flux analysis (Figs. S1 and 2I). Together, these results demonstrated that the most common and significant metabolic changes in both RS and ROIS were the down-regulated lipid metabolism.

### Key genes and metabolites related to senescent cell metabolic changes

Based on the common metabolic changes and pathway alterations between RS and ROIS, we focused on the common key genes and metabolites that modulate the metabolic changes among these two types of senescent cells. We first performed an *in silico* all-against-all gene and metabolite knockout analysis for each study using our developed method FastMM [25], and then performed meta-analysis to obtain the common key genes and metabolites related to metabolic changes in senescent cells.

**Figure 3.**
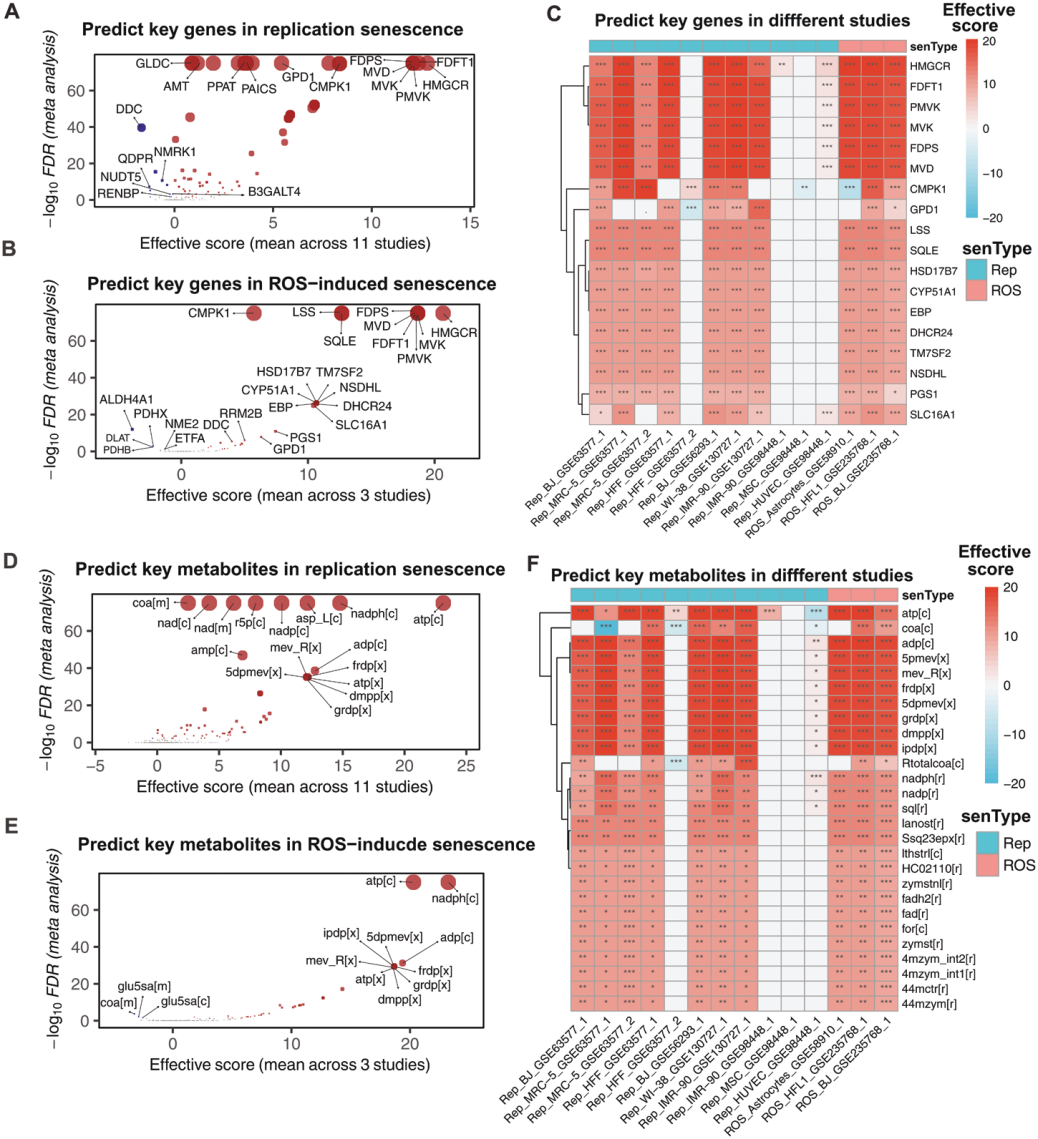
**Predicting key genes and key metabolites by all-against-all knock-out analysis.** (A, B) Predicted key genes in RS (A) and ROIS (B). *X*-axis represents the averaged effective score across the studies, while *Y*-axis represents the −log_10_ FDR from the meta-analysis result using the metDE package in R. (C) Predicted key genes for each study in RS and ROIS. The FDR of < 0.05, < 0.01, and < 0.001 are represented by *, **, and ***, respectively. (D, E) Predicted key metabolites in RS (D) and ROIS (E). (F) Predicted key metabolites for each study in RS and ROIS. The FDR of < 0.05, < 0.01, and < 0.001 are represented by *, **, and ***, respectively.

For key genes, we identified 116 agonist and 12 antagonist key genes in RS, whose overexpression and downregulation, respectively, can potentially rescue or partially rescue the metabolic changes (FDR < 0.05). We found that the enzymes involved in the mevalonate pathway, including HMGCR, FDFT1, PMVK, MVK, FDPS, and MVD, were the most significant agonist genes related to the metabolic changes in senescent cells (effective score > 10, FDR < 1e−60, Figs. 2I and 3A). For ROS-induced senescence, we predicted 60 agonist and 6 antagonist key genes (FDR < 0.05, Fig. 3B). Interestingly, we also observed that the top-ranked key genes in RS were also the top-ranked key genes in ROIS (Fig. 3B). These results were further supported by the individual study analysis that 7/11 and 3/3 studies identified these gene targets in RS and ROIS, respectively (Fig. 3C).

Similarly, for key metabolites, we identified 91 agonist and 1 antagonist key metabolites in RS, whose increased and decreased production, respectively, can potentially rescue or partially rescue the metabolic changes. We found that the metabolites involved in the mevalonate pathway, including 5-phosphomevalonate (5pmev), mevalonate (mev_R), farnesyl-diphosphate (frdp), mevalonate-5-diphosphate (5dpmev), geranyl diphosphate (grdp), dimethylallyl pyrophosphate (dmpp), and isopentenyl diphosphate (ipdp), were the most significant agonist metabolites related to the metabolic changes in replicative senescent cells (effective score > 10, FDR < 1e−20, Fig. 3D). For ROIS, we predicted 35 agonist and 1 antagonist key metabolites (FDR < 0.05). Interestingly, we also observed that the top-ranked key metabolites in RS were also the top-ranked key metabolites in ROIS (Fig. 3E). Consistently, these key metabolites were also supported by the individual study analysis (Fig. 3F).

To further strengthen our conclusions, we incorporated metabolomic data to complement the metabolic modeling results. Through a comprehensive literature review, we identified relevant metabolomic studies for the senescence models analyzed. Specifically, a study by Hu et al. provided metabolomic data for MSCs undergoing replicative senescence [28], which we have re-analyzed and found significant downregulation of mevalonate (log_2_FC = −1.70, *P* = 0.002; Fig. S2). In addition, Tighanimine et al. provided metabolomic data on WI38 fibroblasts during senescence, revealing significant changes in lipid metabolism [29], including the accumulation of triacylglycerol (log_2_FC = 1.56, *P* = 0.008; Fig. S3), further aligning with our observations of impaired lipid metabolism.

### Time series analysis and metabolomics validation for senescent metabolic changes

**Figure 4.**
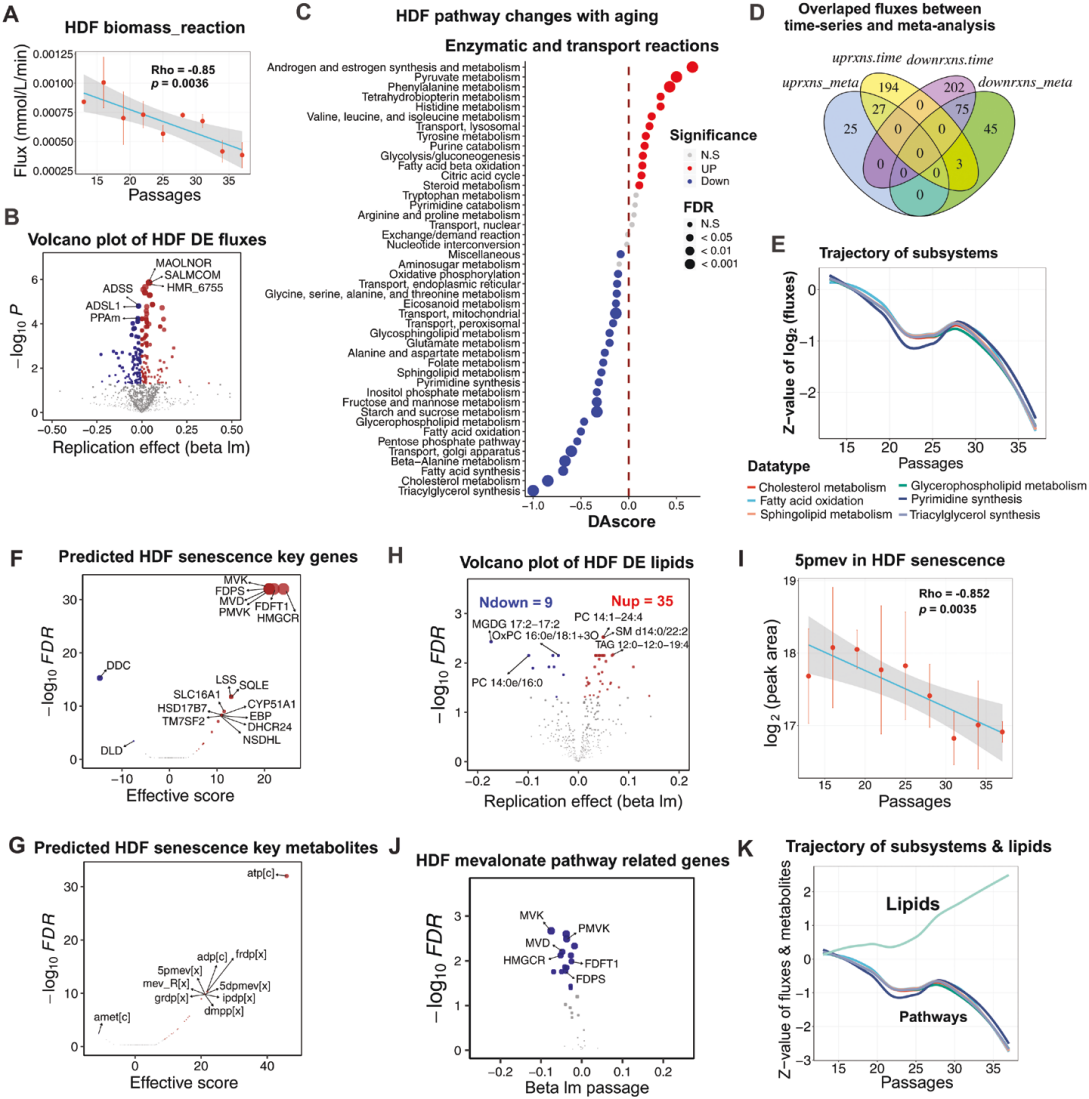
**Time series and metabolism validate in HDF cell line.** (A) Biomass_reaction dynamics from passage 13 to passage 37 (9 points, 2 replications for each point). Rho is −0.85 with a *P*-value of 0.0036. (B) Volcano plot of significant changes in fluxes using time-series analysis. We used the linear model to conduct the time-series analysis (fluxes ~ passages). Fluxes with FDR < 0.05 and beta > 0 or beta < 0 are considered significantly upregulated or downregulated with increasing passages, respectively. (C) Pathway analysis for HDF cellular senescence. (D) Overlapped significant fluxes between time-series analysis and meta-analysis. (E) Representative pathway trajectories in HDF replication senescence. (F, G) Predicted key genes (F) and key metabolites (G) using all-against-all knock-out analyses. (H) Volcano plot of lipid changes in HDF from passage 13 to passage 37. (I) 5pmev level dynamics from passage 13 to passage 37 (9 points, 2 replications for each point) in HDF. Rho is −0.852 with a *P*-value of 0.0035. (J) Volcano plot of mevalonate pathway related gene expression using time-series analysis. We used the linear model to conduct the time-series analysis (gene expression ~ passages). (K) Combined the representative flux pathway trajectories with the up-regulated lipid trajectory in HDF replication senescence.

In addition to the case-control studies, we also examined the trajectories of cellular senescence using time series data. We sequenced the primary dermal fibroblast (HDF) mRNA at 9 time points (13, 16, 19, 22, 25, 28, 31, 34, and 37 passages), each with two replicates [30]. Using these data, we performed precision metabolic modeling analysis using the GPMM method. The results showed that the biomass synthesis abilities gradually decreased by about 3-fold from passage 13 to passage 37 as the cellular passages increased (Fig. 4A, rho = −0.85, *P* = 0.0036). We identified 52 and 123 fluxes that increased and decreased, respectively, with the increase of cellular passages (Fig. 4B). The enrichment analysis showed that the lipid metabolism pathways, such as triacylglycerol synthesis, fatty acid oxidation, cholesterol metabolism, glycerophospholipid metabolism, sphingolipid metabolism, and glycosphingolipid metabolism, were down-regulated along with cellular senescence (Fig. 4C, FDR < 0.05), which was consistent with the case-control meta-analysis.

Next, we compared the significant fluxes between the case-control meta-analysis and the time series analysis. The results showed that in up-regulated reactions, over 50% (27 of 52) of the reactions found in meta-analysis were also up-regulated in our time-series study, while 61% (75 of 123) of the down-regulated fluxes were also significantly down-regulated in meta-analysis (Fig. 4D), further supporting the reliability of the meta-analysis. Interestingly, the trajectory of the significant metabolic changes was not linear and displayed a stable stage between passage 22 and passage 28 (Fig. 4E), indicating the nonlinear nature of metabolic changes in cellular senescence.

We also performed the all-against-all knockout analysis and found that, consistent with the meta-analysis results, the most significant genes were the agonist genes related to the mevalonate pathway, including *HMGCR*, *FDFT1*, *PMVK*, *MVK*, *FDPS*, and *MVD* (Fig. 4F, effective score > 10, FDR < 1e−30). Beside adenosine triphosphate (atp), the most significant metabolites were the agonist metabolites including 5pmev, mev_R, frdp, 5dpmev, grdp, dmpp, and ipdp, further supporting the consistent metabolic changes and key genes in RS and ROIS (Fig. 4G, effective score > 10, FDR < 1e−20).

To validate the results of precision metabolic modeling, we obtained the metabolomics data using LC/MS in the same HDF passage points from passage 12 to passage 37. Since the most significant metabolic change was the down-regulation of lipid metabolism, we expected that the lipids would accumulate with the cell replication, and thus focused on the lipid changes along with the cellular senescence. Using the lipid database, we identified 398 lipids with the MS2 score > 0.6 (see Methods). Among these lipids, we found 44 significantly changed lipids (Fig. 4H, FDR < 0.05). Notably, we observed that 80% (35 of 44) of the significant lipids were up-regulated while only 20% (9 of 44) lipids were down-regulated (Fig. 4H). Although we fail to detect the mevalonate by using LC/MS technology, we found the phosphate mevalonate, namely 5pmev are significant gradually down-regulated with cell senescence (rho = −0.852, *P* = 0.0035; Fig. 4I). Further, all key genes identified through meta-analysis and time series analysis—namely *HMGCR*, *FDFT1*, *PMVK*, *MVK*, *FDPS*, and *MVD*—exhibit significant downregulation in senescent HDF cell lines (Fig. 4J). Notably, the trajectory of these significant metabolic pathways is related with the observed trend of lipid accumulation (Fig. 4K). These results showed that lipids were gradually accumulated, further supporting our metabolic modeling results of decreased lipid metabolism.

### Decreased lipid metabolism in human tissue aging

**Figure 5.**
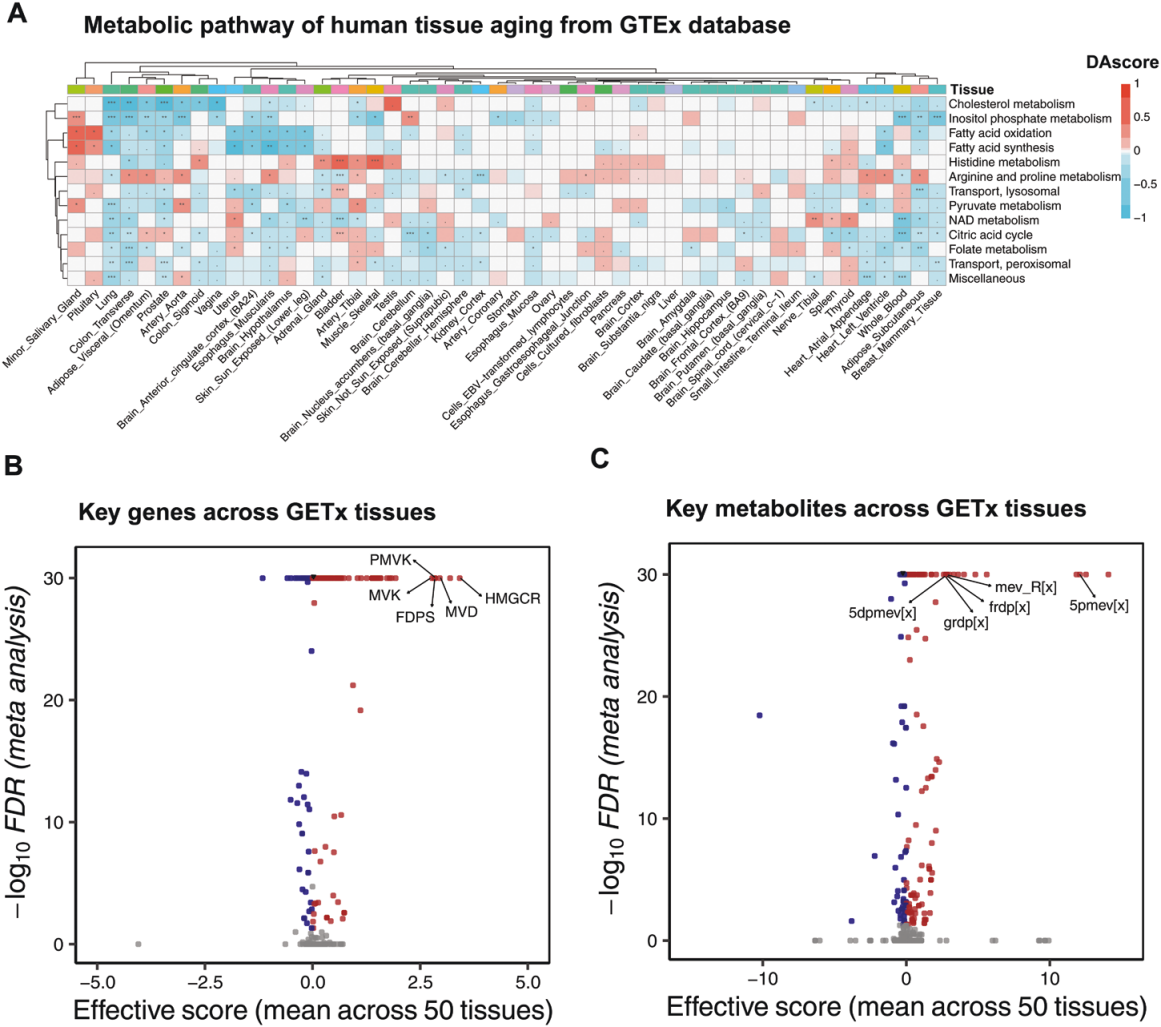
**Metabolic profile of human tissue aging from genotype-tissue expression (GTEx).** (A) Heatmap of consistent metabolic pathway profiles of tissue aging across 50 tissues from GTEx database. The FDR of < 0.05, < 0.01, and < 0.001 are represented by *, **, and ***, respectively. (B, C) Volcano plot of predicted key genes (B) and key metabolites (C). The mevalonate-related genes and metabolites are labeled.

Given the pivotal role of cellular senescence in tissue aging, we hypothesized a corresponding decline in lipid metabolism during human aging. To evaluate this, we performed metabolic modeling on 50 tissue types from the GTEx database, comparing metabolic profiles between adult (aged 30–39, 1317 samples) and elderly (aged 60–69, 5736 samples) groups. The result showed that nearly all lipid-related biosynthesis and consumption pathways are significantly downregulated, including androgen and estrogen synthesis, cholesterol metabolism, inositol phosphate metabolism, sphingolipid metabolism, glycerophospholipid metabolism, and glycosphingolipid metabolism (Fig. S4). These findings highlight a general decline in lipid metabolism-related pathways with aging across various tissues. Notably, pathways such as cholesterol metabolism (meta FDR < 1e−30), fatty acid oxidation (meta FDR = 6.34e−8), fatty acid synthesis (meta FDR < 4.49e−9), and the citric acid cycle (meta FDR < 1e−30) exhibited significant declines (Fig. 5A).

Furthermore, we identified key genes and metabolites implicated in human tissue aging. The genes associated with the mevalonate pathway, which were highlighted in cellular senescence—including *HMGCR*, *PMVK*, *MVK*, *FDPS*, and *MVD*—were also prominent in the aging tissues (meta FDR < 1e−30) (Fig. 5B). Similarly, key metabolites linked to the mevalonate pathway, such as 5pmev[x], mev_R[x], frdp[x], 5dpmev[x], and grdp[x], were identified as significant agonist metabolites in tissue aging (meta FDR < 1e−30) (Fig. 5C). These observations suggest that diminished lipid metabolism and mevalonate pathway dysfunction are shared characteristics of both replicative/ROS-induced cellular senescence and human tissue aging.

### Mevalonate increases the lifespan in *C. elegans*

**Figure 6.**
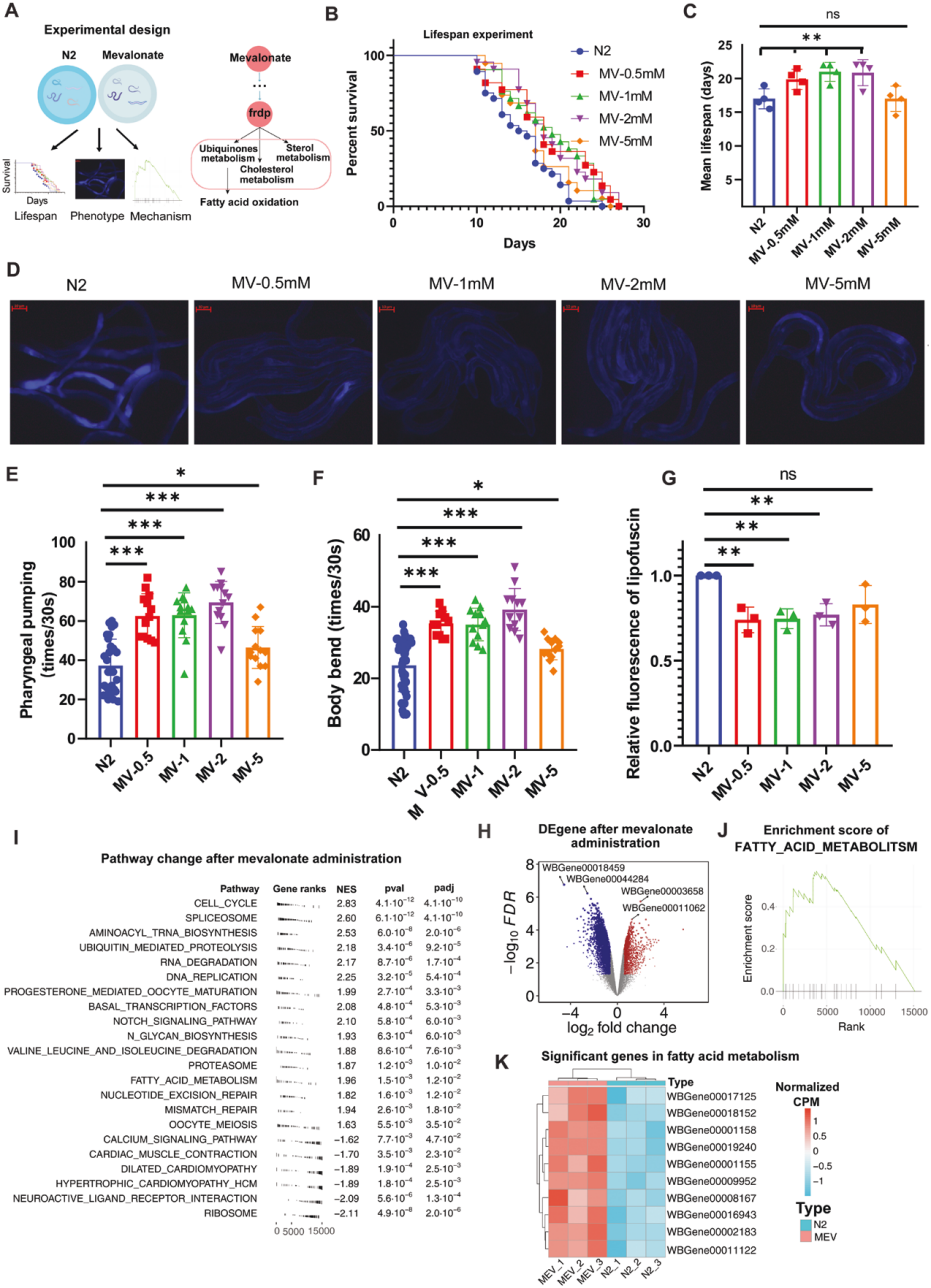
**Mevalonate increased the lifespan and alleviated aging-related phenomena in *C. elegans*.** (A) Schematic of mevalonate in *C. elegans*. (B) Lifespan analysis of N2 and mevalonate (0.5 M, 1 M, 2 M, and 5 M). Lifespan was calculated using the log-rank (Kaplan–Meier) method. (C) Mean lifespan of Fig. 6B (*n* = 4 biological replicates). (D) Fluorescence of lipofuscin (scale bar, 100 μm). Lipofuscin accumulation was measured by blue excitation light (from left to right are N2, 0.5 M, 1 M, 2 M, and 5 M of mevalonate). (E) Pharyngeal pumping assay (*n* = 10/groups for 3 biological replicates). (F) Body bending assay (*n* = 10/groups for 3 biological replicates). (G) Lipofuscin accumulation assay (*n* = 3 biological replicates). Statistical comparisons were performed *t*-test (Fig. 6C, 6E, 6F and 6G). *P*-values of < 0.05, < 0.01, and < 0.001 are represented by *, **, and ***, respectively. ns indicates not significant in *t*-test. (H) Volcano plot of gene expression profiles after mevalonate administration. Up-regulated and down-regulated genes (|log_2_FC| > 0.58 and FDR < 0.05) are colored red and blue, respectively. (I) Gene set enrichment analysis (GSEA) after mevalonate administration. Top 15 up-regulated and down-regulated enrichment pathways are shown. (J) Enrichment plot of FATTY_ACID_METABOLISM in KEGG database. (K) Heatmap of gene expression of significantly changed genes in fatty acid metabolism.

Based on the meta-analysis, our time series data and the tissue aging analysis from GTEx database, we predicted that the metabolites in the mevalonate pathway were the key agonist metabolites that might rescue the metabolic changes in both senescent cells and human tissue aging, thus having the potential for anti-aging or lifespan extension in animal models. To validate our predictions, we selected the key upstream metabolite mevalonate for experimental validation in the widely used animal model *C. elegans* (Fig. 6A), given that mevalonate plays a central role in the mevalonate pathway as a precursor for the biosynthesis of nearly all downstream metabolites, such as 5pmev, 5dpmev, ipdp, frdp, and various isoprenoids.

We administered mevalonate to *C. elegans* at different concentrations and compared the lifespan and the main aging-related phenotypes of nematodes (including phaeopsin abundance, pharyngeal pump movement and worm body swing frequency) between the mevalonate administrate groups and the control group. The results showed that mevalonate significantly increased the lifespan of nematodes at concentrations of 0.5 mM, 1 mM, and 2 mM (*P*-values were 0.0146, 0.0159, and 0.0134, respectively) (Fig. 6B and 6C). In addition, we observed that mevalonate significantly reduced phaeopsin and increased pharyngeal pump movement and body swing frequency (Fig. 6D–G). These results suggest that mevalonate extended the average lifespan of nematodes and improved the aging-related phenotypes of *C. elegans*.

We next performed RNA sequencing to evaluate the potential underlying mechanism of mevalonate on lifespan and health span extension in *C. elegans*. The results showed that 1538 genes were upregulated and 2804 genes were down-regulated after mevalonate administration (Fig. 6H, FDR < 0.05, |log_2_FC| > 0.58). These significantly changed genes were enriched in cellular proliferation related pathways, such as cell cycle, tRNA biosynthesis, DNA repair, and Notch signaling pathway (Fig. 6I). Specifically, we observed that the fatty acid metabolism was significantly upregulated with an enrichment score (ES) of 1.95 (adjusted *P*-value of 0.016) (Fig. 6I and 6J). Notably, we observed that 10 fatty acid metabolism related genes were significantly upregulated after mevalonate administration in *C. elegans* (Fig. 6K). These results were consistent with our prediction that mevalonate could extend lifespan in *C. elegans* by upregulating fatty acid oxidation, at least partially.

## Discussion

**Figure 7.**
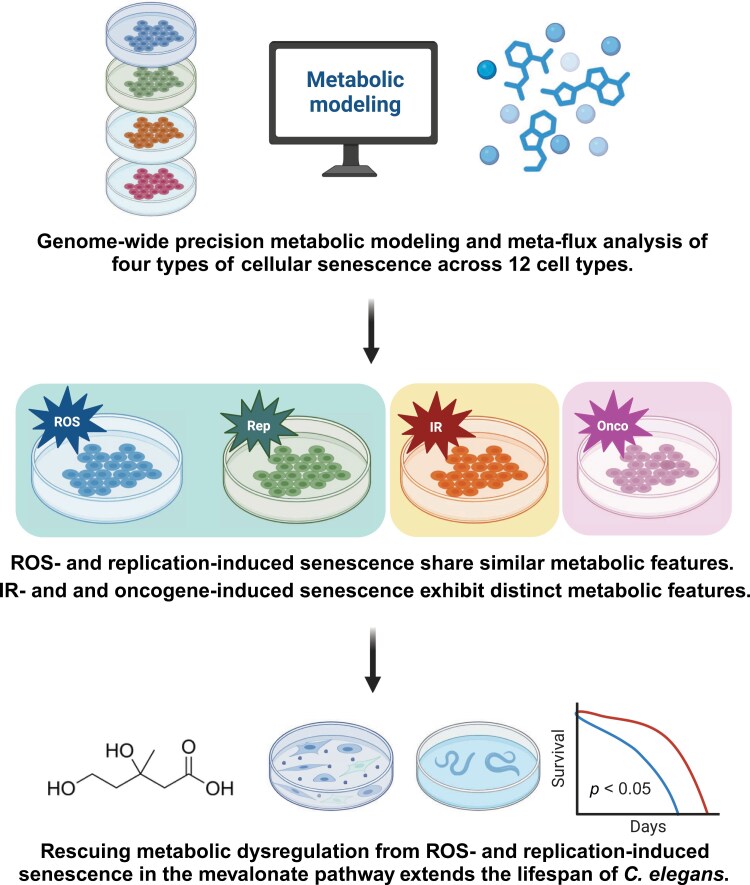
**Graphical summary.** This study employed GPMM and meta-flux analysis to investigate senescence-associated metabolic changes across 12 cell lines representing four types of cellular senescence: replicative (Rep), irradiation-induced (IR), ROS-induced, and oncogene-induced. The results revealed that RS and ROIS share similar metabolic features, characterized by dysregulated lipid metabolism and the mevalonate pathway. In contrast, IIS and OIS exhibit distinct metabolic features. Experimental validation in HDF cell lines and *C. elegans* models supported these findings. Rescuing metabolic dysregulation caused by ROIS and RS in the mevalonate pathway extends the lifespan of *C. elegans*. This study highlights the metabolic heterogeneity and commonalities across different types of senescence, providing valuable insights for translational applications in aging and senescence research. This graphical summary was created using BioRender with the publication license ID of PH27RO8LR0.

In this study, we used genome-wide metabolic modeling and meta-analysis to explore the metabolic changes in four types of cellular senescence (replicative, irradiation-induced, ROS-induced, and oncogene-induced) across 12 cell lines. We revealed the common metabolic features in replication and ROS-induced cellular senescence, but heterogeneity in irradiation and oncogene-induced senescence. We found that the replicative and ROS-induced cellular senescence were associated with profound metabolic changes in lipid metabolism, and that these changes could be modulated by key genes and metabolites in the mevalonate pathway. We demonstrated that administration of mevalonate, an upstream metabolite in the mevalonate pathway, could rescue the metabolic dysfunction and extend the lifespan of nematodes (Fig. 7). Our findings provide a comprehensive insight into the metabolic landscape of cell senescence and suggest potential anti-aging interventions.

Metabolic modeling offers distinct advantages over traditional analyses in discerning metabolic changes [31–36]. This approach is adept at capturing subtle metabolic signatures that may be obscured by the broader spectrum of changes, often referred to as “noise.” For instance, our prior research on centenarian metabolism revealed an upregulation of fatty acid oxidation as a prominent metabolic feature in centenarians—a signature not readily detected by conventional methods [26]. Despite the upregulation of most fatty acid oxidation-related genes, their changes are not always top-ranked or statistically significant, causing this signature to be masked by other, more prominent changes [26]. Similarly, our current study underscores the benefits of metabolic modeling in analyzing cellular metabolism. Conventional methods, such as GO enrichment via Metascape [37] or GSEA [38, 39], struggled to identify significant shifts in lipid metabolism during replication-induced senescence (Figs. S5 and S6). The challenge lies in the metabolic signature being intermingled with a plethora of other alterations. However, upon examining genes associated with lipid metabolism, we observed a predominant downregulation of genes with significant changes (meta FDR < 0.01, Fig. S7). Remarkably, all identified significant genes involved in cholesterol metabolism (6 out of 6), fatty acid oxidation (15 out of 15), and a majority of glycerophospholipid metabolism genes (12 out of 20) exhibited downregulation. Recent metabolic studies in animal models have shown decreased fatty acid oxidation and increased lipid accumulation with aging [40, 41], which provided additional support to our observations and argued for the efficacy of metabolic modeling in unveiling hidden signatures within cellular metabolism studies.

Understanding the metabolic profiles of cellular senescence is important for elucidating the metabolic mechanism of this process. However, it is still unclear whether different types of cellular senescence have common metabolic features or not. Some studies showed their common gene expression profiles in RS [42], while others showed heterogeneity in different types of senescence [43]. Here, we revealed the common metabolic features between RS and ROIS, mainly the decreased lipid metabolism and the accumulation of cellular lipids in senescent cells. These findings suggest that lipid metabolism is a key metabolic feature of senescence induced by replication or oxidative stress. On the other hand, for IIS and OIS, we did not observe the decreased lipid metabolism and, conversely, 3/4 cholesterol metabolism pathways were increased (Fig. 2D and 2F) in OIS. These results indicate that senescence induced by DNA damage or oncogenic stress has distinct metabolic profiles from senescence induced by replication or oxidative stress. These results provide a systematic view of cellular senescence and reveal the metabolic diversity and complexity of this phenomenon.

Our study reveals that RS and ROIS share similar metabolic alterations, while IIS and OIS exhibit more diverse and distinct metabolic profiles. Both RS and ROIS are marked by a significant decline in lipid metabolism and reduced activity in the mevalonate pathway, likely driven by oxidative stress—an essential factor in both senescence and metabolic reprogramming [44]. RS is characterized by the accumulation of cellular damage over successive replications, leading to a gradual increase in ROS production due to mitochondrial dysfunction [45, 46]. Similarly, ROIS is directly induced by oxidative stress, imposing a metabolic burden on cells that mirrors the effects observed in RS [47]. Our data support the hypothesis that this oxidative burden contributes to reduced fatty acid oxidation and impaired lipid synthesis, as both RS and ROIS exhibit substantial declines in cholesterol metabolism and sphingolipid pathways (Fig. 2G and 2H). Furthermore, PCA clustering of RS and ROIS flux distributions (Fig. 2E) suggests that oxidative damage induces similar metabolic reprogramming across these senescence types. Collectively, these findings underscore the role of oxidative stress not only as a consequence of cellular aging but also as a critical driver of metabolic dysfunction in senescent cells.

Differently, the heterogeneity observed in DNA damage responses to irradiation across both cancerous and normal cells suggests that various studies may report disparate underlying mechanisms [48, 49]. Our extended analysis of genes associated with cell growth and oncogenic profiles in senescent cells revealed a significant downregulation of oncogenes (80%) in both RS and ROIS (Fig. S8). However, this pattern was not as pronounced in IIS and OIS, where only approximately half of the significantly altered oncogenes were downregulated (53% for IIS and 58% for OIS) (Fig. S8). These findings elucidate why IIS and OIS are metabolically distinct from RS and ROIS. Furthermore, they explained why our method was able to detect cell growth arrest in RS and ROIS, but not in IIS and OIS (Fig. 1D–G). At the gene expression level, RS and ROIS exhibit more profound cell growth arrest signaling compared to IIS and OIS. This differential signaling intensity may explain why signals of cell growth arrest in RS and ROIS, rather than IIS or OIS, were captured by our method.

Different from some studies that predicted distinct key gene/metabolite profiles between RS and ROIS, our meta-analysis showed RS and ROIS share some similarities in metabolic changes (Fig. 3C and 3F). This difference is likely attributed to factors like smaller passage differences or different RNA-seq methods. For example, “HFF_GSE63577_2” had a smaller passage range (18 passages) than other studies (> 25 passages), possibly causing subtler changes. “IMR-90_GSE98448_1” used two RNA-seq types (“polyA” and “nascent”), leading to potential inconsistencies. “MSC_GSE98448_1” studied MSCs, whose multipotent differentiation properties may account for their distinct profiles.

We propose the mevalonate pathway as a key modulator of the metabolic changes in RS and ROIS and in human tissue aging. The mevalonate pathway is a key lipid biosynthetic and metabolic pathway that produces cholesterol, isoprenoids, and dolichols, which are essential for various cellular functions, such as membrane integrity, protein prenylation, and glycosylation [50]. Moreover, the mevalonate pathway also synthesizes essential mitochondrial cofactors, such as coenzyme Q10 and other ubiquinone metabolites, and heme. These products are essential for mitochondrial function and lipid oxidation, which are important aspects of cellular metabolism [51]. Contrary to the conventional view that inhibiting the mevalonate pathway would reduce obesity, recent evidence indicates that mevalonate pathway inhibitors actually promote obesity by impairing adipocyte oxidation in humans and mice [52–54]. Previous studies also demonstrated that the mevalonate pathway is disrupted in senescent cells and that inhibition of this pathway can trigger senescence [55]. Disruptions in the mevalonate pathway have been implicated in the development of several age-related diseases, including cardiovascular diseases due to altered cholesterol metabolism, and neurodegenerative diseases where protein prenylation plays a role in neuronal function [55, 56]. These studies support our finding that the mevalonate pathway was down-regulated in both replicative and ROIS, and that administration of mevalonate, an upstream metabolite in this pathway, could rescue the metabolic dysfunction and extend the lifespan and health span of *C. elegans*. We also found that studies in other species have shown similar changes in the mevalonate pathway during aging. For instance, a study from Liu et al. observed decreased levels of mevalonate and its derivatives in aged mouse tissues, linking mevalonate pathway abnormalities to oocyte meiotic defects and aneuploidy [57]. In humans, Zhang et al. also demonstrated that mevalonate levels decrease with male reproductive aging, indicating that the downregulation of this pathway is pertinent to human senescence as well [55].

Through comprehensive all-against-all knockout analysis, we identified key regulators implicated in the dysfunction of the mevalonate pathway and lipid metabolism. We discovered that the key genes (namely, HMGCR, FDFT1, PMVK, MVK, FDPS, and MVD), which are integral to the mevalonate pathway and lipid metabolism, are consistently associated not only with cellular senescence (as shown in Figs. 3A, 3B and 4F) but also with aging in human tissues (Fig. 5B). Indeed, these genes were found to be significantly downregulated during cellular senescence (Fig. 4J), partially explaining the reason why mevalonate decreases during aging. These findings suggest that these genes may underlie the mechanisms contributing to cellular senescence and tissue aging. Previous studies have indicated that silencing the MVK gene via RNA interference reduced the median lifespan by 14% in wild-type and by 33% in *daf-2* mutants of *C. elegans* [58, 59], which partially corroborates our results. However, the direct causal relationship between enhanced lipid metabolism due to mevalonate loading and lifespan extension warrants further investigation in future studies. As a precursor to ubiquinone (coenzyme Q), mevalonate is essential for mitochondrial electron transport and energy production [50]. Enhanced mitochondrial function may reduce oxidative stress and delay senescence. While the direct regulation of fatty acid oxidation by mevalonate in *C. elegans* is complex, our evidence obtained so far suggests that mevalonate supplementation increases the expression of genes involved in fatty acid oxidation, which may regulate cellular senescence, although further validation is needed.

In conclusion, our study provides a comprehensive and systematic view of the common and heterogeneous metabolic changes during cell senescence and identifies the mevalonate pathway as a key modulator and potential target for anti-aging interventions. Our findings open new avenues for further research on the metabolic aspects of senescence and aging and suggest novel strategies to improve the health and longevity of aging populations.

## Research limitations

Our study also has some limitations that should be acknowledged and addressed in future research. We utilized genome-scale metabolic modeling to integrate transcriptomic data and predict metabolic flux distributions. This approach, based on gene–protein reaction (GPR) associations, allows us to estimate metabolic states and has been validated in various biological contexts [35]. Nevertheless, additional metabolomic data would be of help to further strengthen these observations. First, although we collected 178 samples from 27 studies for metabolic modeling and meta-analysis, we may have missed some relevant studies, and the number of ROIS and OIS studies was relatively small, which may introduce bias and reduce the generalizability of our findings. Second, our experimental validation was performed in *C. elegans*, which is a useful model organism for aging research, but may not fully capture the complexity and diversity of cellular senescence in other organisms. Therefore, further studies are needed to validate and extend our findings in other model systems, and to explore the molecular mechanisms and pathways that mediate the common and heterogeneous features of different types of cellular senescence. Third, the underlying molecular interactions between the dysfunctional mevalonate pathway and cellular senescence demand deeper exploration. For instance, examining the influence of mevalonate on pivotal signaling pathways that are known to regulate aging in animal models, such as the insulin/insulin-like growth factor-1 signaling axis, and its effects on transcriptional and epigenetic regulation is essential. Although we performed RNA-seq analysis of *C. elegans*, it is still difficult to conduct systems modeling on this animal due to the absence of a fully annotated general metabolic map for *C. elegans*. We hope that in near future, our genome-wide precision metabolic modeling methods can be accurately applied to more animal models, including *C. elegans*, when their annotated metabolic maps are available.

## Methods

### Research ethics

There are no relevant ethical issues.

### Dataset collection

We searched for studies on cellular senescence and gene expression in GEO and EBI databases using two keywords: “cell senescence” and “gene expression.” We also used the citation network from a hotspot study entitled “Unmasking transcriptional heterogeneity in senescent cells” [43] to find relevant studies. We applied the following criteria to select the studies: (i) The dataset is high-throughput RNA sequencing; (ii) The dataset has at least two control (proliferative) samples and two case (senescent) samples; (iii) The case samples and the control samples within each study have the same platform, sample RNA source and sequencing technology to avoid technical bias. Finally, we collected 27 studies on four types of cellular senescence (replicative, irradiation-induced, ROS-induced, and oncogene-induced) across 12 cell lines in 10 public RNA-sequencing datasets (Fig. 1B). Since the data from these 27 studies were from different platforms (GPL11154, GPL16791, GPL21697), different RNA sources (total RNA and mRNA), and different sequencing technologies (single-end and paired-end), there was no perfect method to deal with these complex situations. We therefore used the processed raw counts or FPKM from the published papers as the authors provided, assuming that the authors from these studies had considered their own data parameters.

### Genome wide precision metabolic modeling and fluxes analyzing

#### Metabolic modeling

We used our recently developed genome-wide precision metabolic modeling method, GPMM, to perform the genome-wide metabolic modeling [26]. Briefly, GPMM integrates the estimated protein abundance from gene expression and enzymatic kinetic parameters into the generic human metabolic model as upper bounds based on Michaelis–Menten kinetics. In addition, the nutrient uptake fluxes of cell lines are derived from the literature [60] and the lower bound of other exchanges was set at zero. The flux variability analysis is conducted to construct the tissue-specific models for each sample using the FastMM algorithm [25]. Since ATP production is essential for all cells, we constrained the low bound of ATP production as 90% of its optimized value, similar to previous studies [61]. Similarly, the lower bound of the fluxes of biomass was also set at 90%.

#### Flux change identification

For case–control design, the flux changes between proliferation and senescent cells were calculated using the Limma package [62] based on the values of metabolic modeling.

#### Meta-analysis of flux changes

The meta-analysis for each senescent type (RS, ROIS, IIS and OIS) was performed using the MetaDE package in R (version 2.2.3) and set the “meta.method” parameter of “FEM.” Fluxes with the FDR < 0.05, and *Z*-value > 0 were consider as significant upregulated fluxes, while fluxes with the FDR < 0.05 and *Z*-value < 0 were consider as significant down-related fluxes. The meta-log_2_ FC was calculated as the averaged log_2_FC among different studies for each senescent type.

#### Identifying metabolic pathway changes

The differential abundance score (DA score) was calculated using the previously published method [27]. For each metabolic pathway (*i*), the DA score ($DA_{i}$) can be calculated as following (Eq. (1)):


$$\begin{array}{l} DA_{\text{i}}=\frac{\#\;up\;regulated\;fluxes-\#\;down\;regulated\;fluxes}{Total\;reactions\;in\;ith\;subsystem} \end{array}$$ (1)

The significance (*P*-values) of DA scores was calculated used a “bootstrap without replacement” method as described in our previously study [26].

#### Meta-analysis of pathway changes

The meta-analysis of pathway changes is also calculated using the differential abundance score as describe above. In this case, the up-regulated and down-regulated fluxes were used as the significant upregulated and down-regulated fluxes from meta fluxes analysis for each SEN types, respectively.

### All-against-all knockout analysis

#### Gene knockout analysis

*In silico* knockout analysis was performed to obtain the effect of each gene knockout on each reaction by using the single gene deletion knockout algorithm [25] and performed in R (see data availability section). We thus obtained an all-against-all gene knockout matrix (*G*^KO^), where rows and columns represented the genes and reactions, respectively. As the flux of transport was not constrained in GPMM due to the lack of enzymatic parameters [26], we removed the knockout results of transporters.

Then, the gene effective score (GES) can be calculated as the following (Eq. (2)):


$$\begin{array}{l} GES_{\text{i}}=\sum_{j=1}^{n}G_{i,\text{j}}^{\left(KO\right)}\;\times \;sign\left(log_{2}FC_{\text{j}}\right)\;\times \;L_{\text{j}}\;\;\; \end{array}$$ (2)

Where $i\;and\;j\;represent\;the\;ith\;gene\;and\;the\;jth\;reaction,\;and\;log_{2}FC_{j}$ and $L_{j}$ are the log_2_ fold change and logical of (significance *P* < 0.05 AND log_2_ > 0.5) in the *j*th flux, respectively.

The significance for each GES for each study was calculated using norm-background method using the “pnorm” function in R. FDR was calculated using the “p.adjust” function using the “FDR” method in R. The value of GES > 0 or < 0 and FDR < 0.05 indicates that knocking out this gene can either rescue or enhance the flux changes in senescence cells, respectively.

#### Meta-analysis of gene knockout analysis

The meta-analysis of gene knockout analysis is also performed using the MetaDE package in R (version 2.2.3) and using the “MetaDE.pvalue” function to calculate the meta *P*-values and FDRs for each SEN type.

#### Metabolite knockout analysis

The metabolite knockout analysis was conducted using the algorithm in FastMM [25] to obtain the all-against-all metabolite knockout matrix(M^(KO)^), where row and column represent the metabolites and reaction, respectively.

Similar to the gene knockout analysis, we obtained metabolite effective score (MES) as follows (Eq. (3)):


$$\begin{array}{l} MES_{\text{i}}=\sum_{j=1}^{n}M_{\text{i},\text{j}}^{\left(KO\right)}\;\times \;sign\left(log_{2}FC_{\text{j}}\right)\;\times \;L_{\text{j}}\;\;\; \end{array}$$ (3)

Where $M_{\text{i},\text{j}}^{\left(EM\right)}$ represented the effect score of the *i*th metabolite on the *j*th reaction. Similar to the gene targets, the significance (*P*-value) for each GES for each studies was calculated using norm-background method using the “pnorm” function in R and the FDR was calculated using the “p.adjust” function using the “FDR” method in R. The value of MES > 0 or < 0 and FDR < 0.05 indicates that knocking out these metabolites can either rescue or enhance the flux changes in senescence cells, respectively.

#### Meta-analysis of metabolite knockout analysis

Similar with the meta-analysis of gene knockout analysis, we also used the MetaDE package in R (version 2.2.3) and using the “MetaDE.pvalue” function to calculate the meta *P*-values and FDRs for each SEN type.

### HDF time series flux analysis

#### RNA sequence data

The HDF cells were seeded and cultured into 96-well plates at a density of 8 × 10^3^. Total RNA samples were extracted using the TRIzol method (Invitrogen). The polyA-enriched RNA-seq libraries were prepared for sequencing using the Illumina HiSeq 4000 platform. We used salmon package (version 1.10.0) to calculate the transcript length and read counts of transcripts using the index of all cDNA in GRCh38 (version 110). We then merged these lengths and read counts into gene length and read counts using the tximport package in R (version 1.26.1). Finally, we calculated the FPKM from the gene length and read counts using the DEseq2 package (version 1.38.3) as the input for genome wide precision metabolic modeling.

#### Time series flux analysis

The fluxes were also performed using the GPMM method [26] as described above. The flux changes among different passages were calculated by using the following linear model (Eq. (4)):


Undefined control sequence \textasciitilde (4)

#### Time series pathway analysis

The overall changed pathway analysis was also used the DAscore as described in Eq. (1). The trajectory of pathway analysis was conducted by the series comparison between start passage (the passage 12) and other passage points. Each comparison was also using the “case-control” method as describe above.

#### Time series knock-out analysis

Gene and metabolite knockout analysis were also used the FastMM method as described above and here the sign of log_2_FC” changed into the sign of beta value of linear model.

### HDF cell line metabolomic analysis

#### Metabolites extraction

1000 μL extract solution (acetonitrile: methanol: water = 2: 2: 1) containing isotopically-labeled internal standard mixture was added to the sample. After 30 s vortex, the samples were homogenized at 35 Hz for 4 min and sonicated for 5 min in ice-water bath. The homogenization and sonication cycle were repeated for three times. Then the samples were incubated at −20°C for 1 h and centrifuged at 12,000 rpm for 15 min at 4°C. The resulting supernatant was transferred to a fresh glass vial for LC/MS analysis. The quality control (QC) sample was prepared by mixing an equal aliquot of the supernatants from all of the samples.

#### LC–MS/MS analysis.

LC–MS/MS analyses were performed using an UHPLC system (Vanquish, Thermo Fisher Scientific) with a UPLC BEH Amide column (2.1 mm × 100 mm, 1.7 μm) coupled to Q Exactive HFX mass spectrometer (Orbitrap MS, Thermo). The mobile phase consisted of 25 mmol/L ammonium acetate and 25 ammonia hydroxide in water (pH = 9.75) (A) and acetonitrile (B). The analysis was carried with elution gradient as follows: 0–0.5 min, 95% B; 0.5–7.0 min, 95%–65% B; 7.0–8.0 min, 65%–40% B; 8.0–9.0 min, 40% B; 9.0–9.1 min, 40%–95% B; 9.1–12.0 min, 95% B. The column temperature was 25°C. The auto-sampler temperature was 4°C, and the injection volume was 2 μL. The QE HFX mass spectrometer was used for its ability to acquire MS/MS spectra on data-dependent acquisition (DDA) mode in the control of the acquisition software (Xcalibur, Thermo). In this mode, the acquisition software continuously evaluates the full scan MS spectrum. The ESI source conditions were set as following: sheath gas flow rate as 50 Arb, Aux gas flow rate as 10 Arb, capillary temperature 320°C, full MS resolution as 60,000, MS/MS resolution as 7500, collision energy as 10/30/60 in NCE mode, spray Voltage as 3.5 kV (positive) or − 3.2 kV (negative), respectively.

#### Data preprocessing and annotation

The raw data were converted to the mzXML format using ProteoWizard and processed with an in-house program, which was developed using R and based on XCMS, for peak detection, extraction, alignment, and integration. Then lipidblast was applied in metabolite annotation. The cutoff for annotation was set at the MS2 score > 0.6.

#### Significant metabolites identification

The peak area of metabolism was log_2_ transformed and using the linear model (metabolites ~ passages) to find the significantly changed metabolites. We considered the metabolites with a beta value > 0 and FDR < 0.05 as significantly upregulated metabolites with cellular replication, and the metabolites with a beta value < 0 and FDR < 0.05 as significantly downregulated metabolites with cellular replication.

### Human tissue aging metabolic modeling and flux analysis

The gene expression of human tissues in different age group were obtained from the GTEx database [63]. To elucidate the metabolic shifts associated with tissue aging, we applied GPMM to the gene expression data (measured in FPKM) for all available human tissues within the GTEx repository, encompassing a total of 50 tissue types. This analysis facilitated a comparative assessment of metabolic fluxes between two age groups: the elderly (aged 60–69, 5736 samples) and adults (aged 30–39, 1317 samples). The methodologies employed for identifying differential fluxes, conducting metabolic pathway analysis, and predicting key genes and metabolites are the same those above described for cellular senescence.

### Experimental validation

#### Lifespan assay

All lifespan experiments in *C. elegans* were conducted at 20°C. Lifespan assay was conducted according to a standard protocol with OP50. In brief, elegans were bleached to get synchronized eggs, these eggs were cultured until breeding, and then, the naturally bred eggs were picked for lifespan assay. At the L4 molt, elegans were transferred to plates containing 20 M 5-fluoro-2’-deoxyuridine (FUDR) and M6 at a final concentration of 0.5 M, 1 M, 2 M and 5 M. The first day of adulthood (L4) was recorded as day 1 for lifespan analysis. The plates were refreshed daily. Initial about 50 elegans were used for each treatment. Dead elegans were counted from day 8. Elegans of no response to nose-touching were picked out of the bacteria lawn, then those motionless elegans were scored as dead. Graphpad Prism 8 were used to analyze lifespan data. *P* values were calculated with the log-rank (Kaplan–Meier) method.

#### Body bending assay

Elegans were pretreated as in lifespan assays. On the 10 day, 100 μL M9 buffer was pipetted onto the surface of an empty NGM plate, one elegan was picked into the buffer, and allowed 30 s to recover from the transfer. Then the number of elegans thrashing during 30 s was counted, four times for each elegan, 15 to 20 elegans for each treatment. One elegan from one treatment was counted, then one elegan from another treatment, then the third treatment, and so on, the cycle was repeated to avoid the interference of environment or operation. The significance of comparisons between the control and M6 groups was calculated by using Student’s *t*-test.

#### Lipofuscin accumulation

Elegans were pretreated as in lifespan assays. The autofluorescence of intestinal lipofuscin was measured as an index of senescence of day 10 adults. Randomly selected elegans from each bacterial lawn were washed three times with M9 buffer. Then, the elegans were placed onto a 2% agar pad coated with 10 mM sodium azide in M9 buffer to be paralyzed. Lipofuscin autofluorescence images were taken using blue excitation light (405−488 nm) with DAPI (4′,6-diamidino-2-phenylindole) and laser confocal scanning microscopy. Fluorescence was quantified using ImageJ software to determine the lipofuscin levels. Three independent experiments were performed with more than 30 elegans for each bacterial species on each day. Lipofuscin levels were quantified by determining average pixel intensity using ImageJ software in each worm’s intestine.

#### Pharyngeal pumping

Elegans were pretreated as in lifespan assays. Pumping rates were assayed on day 10 of adulthood. Pumps of the terminal pharyngeal bulb were counted for 30 s intervals per elegans crawling on the bacterial lawn. Pumping rates per min for 10 elegans per treatment were averaged. Pumping rates were assessed in three independent replicate experiments. The significance of comparisons between the control and M6 groups was calculated by using Student’s *t*-test.

### RNA-sequencing analysis of *C. elegans*

Total RNA samples were also extracted using the TRIzol method (Invitrogen). And, the polyA-enriched RNA-seq libraries were also prepared for sequencing using the Illumina HiSeq 4000 platform. Salmon software (version 1.10.0), trimport package (version 1.26.1) were also used to obtain the raw counts of gene expression of *C. elegans*. Normalized counts per million reads mapped (CPM) was calculated using the trimmed mean of M values (TMM) method in the edgeR package for next step analysis [64], as a comparative analysis showed that the method of normalized raw counts is more robust for differential gene expression analysis then other methods (such as fpkm or TPM) [65, 66]. The differential gene expression was performed using the Limma package based on the normalized CPM. The GSEA analysis was performed by using the fgsea package in R [38].

### Statistics analysis

The statistical methods and tools used for metabolic modeling, flux analysis, key gene and metabolite identification, differential gene expression analysis, and meta-analysis are explicitly described. All analyses were performed using R. Experiments were conducted at least three times, and a one-way *t*-test *P*-value of < 0.05 was considered statistically significant.

## Supplementary Material

lnaf003_suppl_Supplementary_Tables_S1-S4

lnaf003_suppl_Supplementary_Figures_S1-S8

## Data Availability

The code and processed data in this manuscript can be available from: github.com/GonghuaLi/Code_for_publications/tree/master/Cell_senescence_metabolism
