# Supplementary material for: Unraveling the metabolic heterogeneity and commonality in senescent cells using systems modeling
Source: Life Med. 2025 Jan 20;4(2):lnaf003. doi: 10.1093/lifemedi/lnaf003 (PMC11992571; doi:10.1093/lifemedi/lnaf003)
Supplement: lnaf003_suppl_Supplementary_Figures_S1-S8 [file lnaf003_suppl_supplementary_figures_s1-s8.docx]

**Unraveling the metabolic heterogeneity and commonality in senescent cells using systems modeling**

Gong-Hua Li^1,#,^*, Yu-Hong Li^2,#^, Qin Yu^1^, Qing-Qing Zhou^1^, Run-Feng Zhang^1^, Chong-Jun Weng^1^, Ming-Xia Ge^1^, Qing-Peng Kong^1,3,^*

^1^Key Laboratory of Genetic Evolution & Animal Models (Chinese Academy of Sciences), Key Laboratory of Healthy Aging Research of Yunnan Province, Kunming Key Laboratory of Healthy Aging Study, Kunming Institute of Zoology, Chinese Academy of Sciences, Kunming 650201, China

^2^College of Biological Resources and Food Engineering, Qujing Normal University, Qujing 655000, China

^3^CAS Center for Excellence in Animal Evolution and Genetics, Chinese Academy of Sciences, Kunming 650223, China

^#^These authors contributed equally to this work.

*Correspondence: kongqp@mail.kiz.ac.cn (Q.-P.K), ligonghua@mail.kiz.ac.cn (G.-H.L)

_
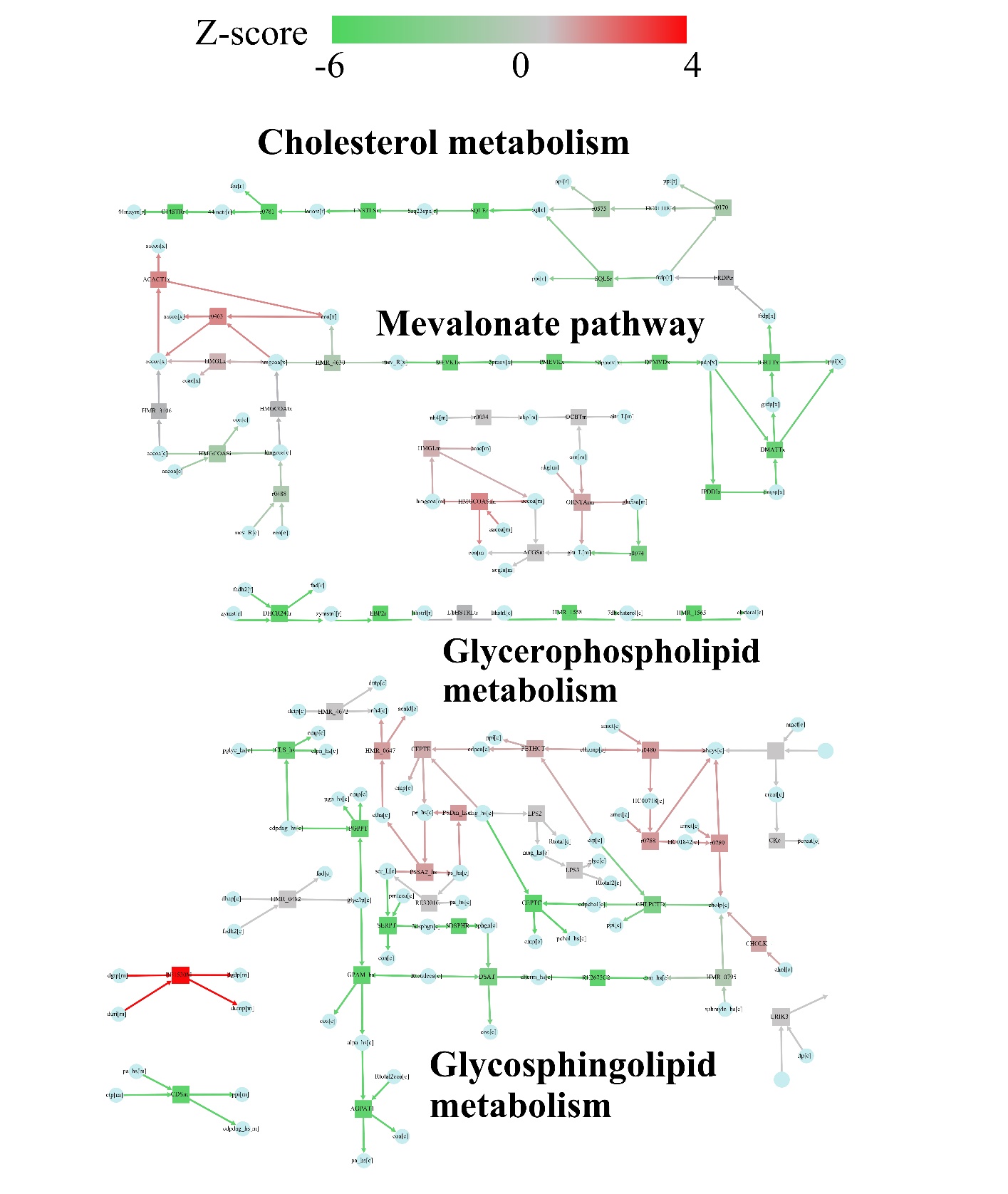
_

**Figure S1.** The most significant pathways that changed in replication and ROS-induced senescence are represented.

Reactions with FDR < 0.05 and *Z*-value < 0 are colored green, and reactions with FDR < 0.05 and *Z*-value > 0 are colored purple.


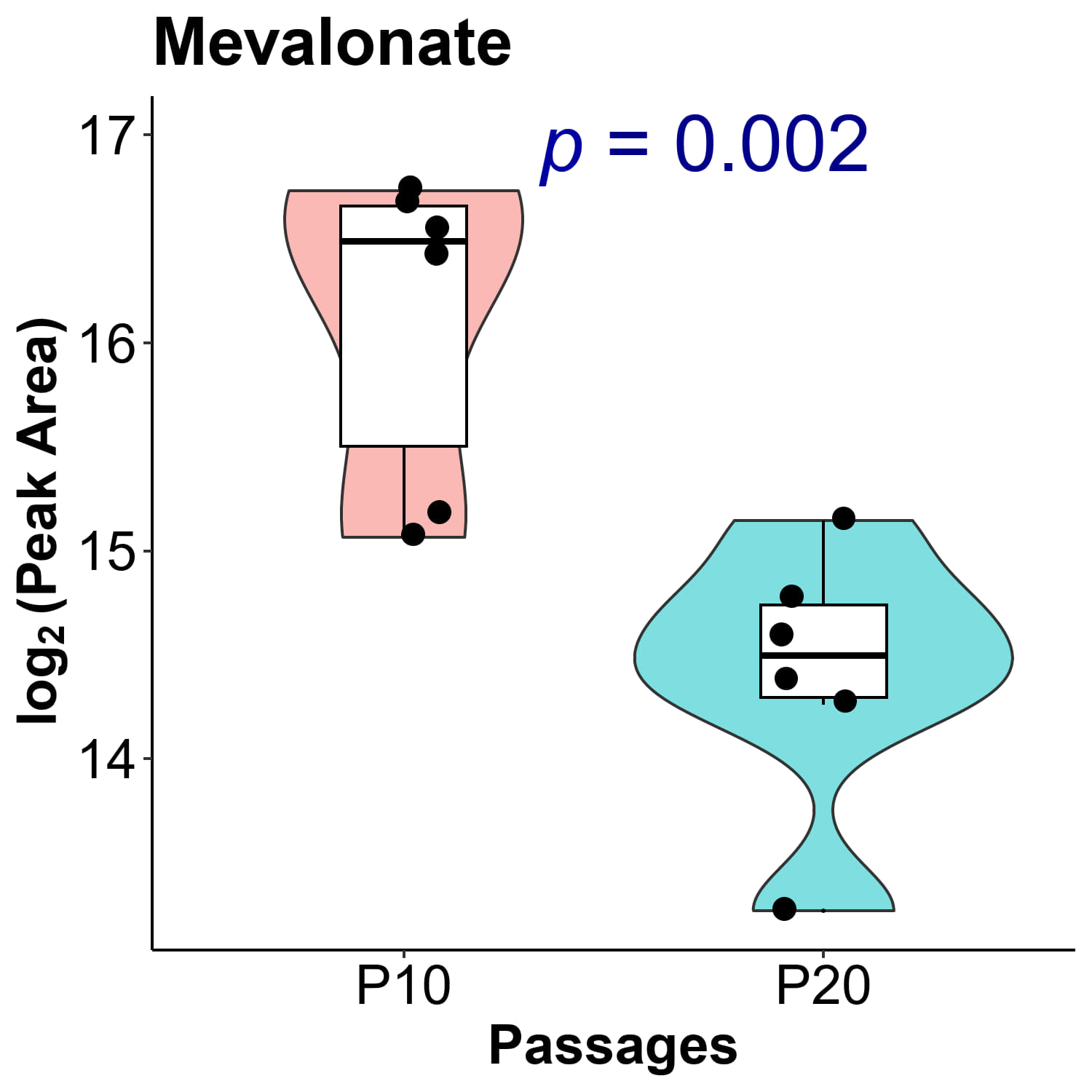


**Figure S2.** Mevalonate is downregulated in MSCs undergoing replicative senescence (data from Hu et al., 2024).


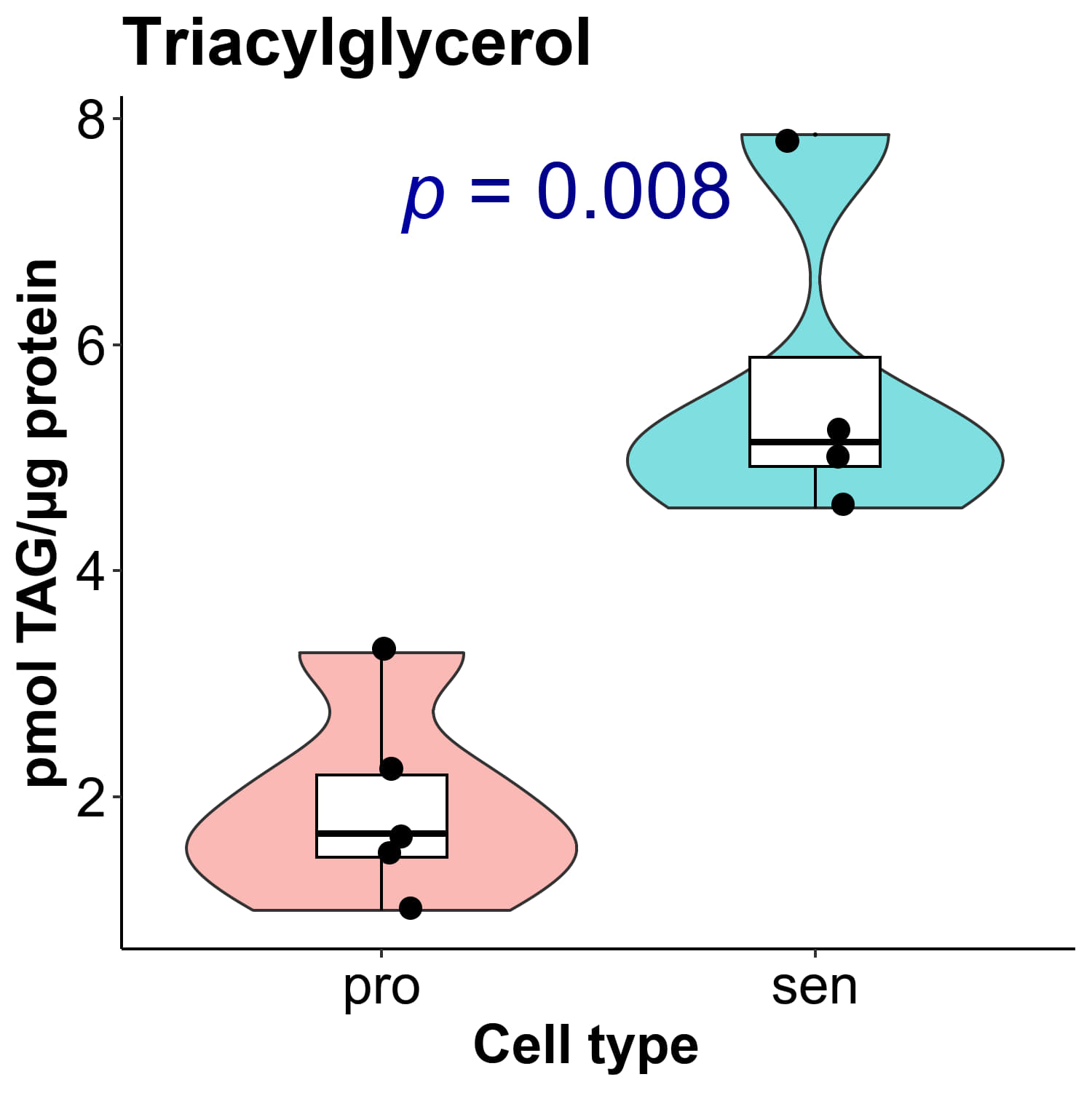


**Figure S3.** Accumulation of triacylglycerol in WI38 fibroblasts during replicative senescence (data from Tighanimine et al., 2024).

Note: “pro” and “sen” represented as proliferative cell and senescent cell, respectively.


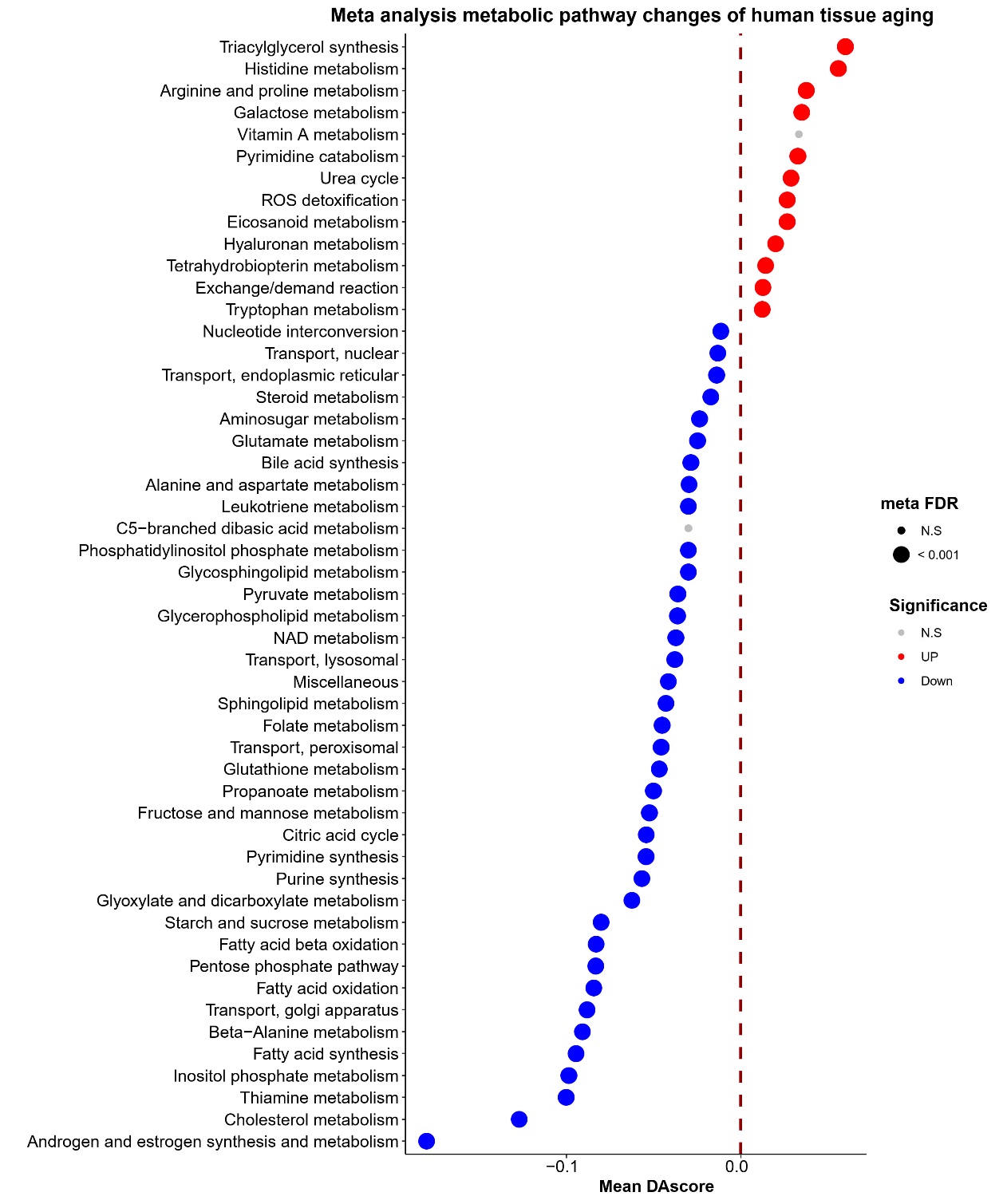


Figure S4. Metabolic pathway changes in human tissues during aging across 50 tissues.

The *x*-axis represents the mean DA score across the 50 tissues, and the *y*-axis represents the metabolic pathway names. Pathways that are significantly upregulated or downregulated are colored in red and blue, respectively.


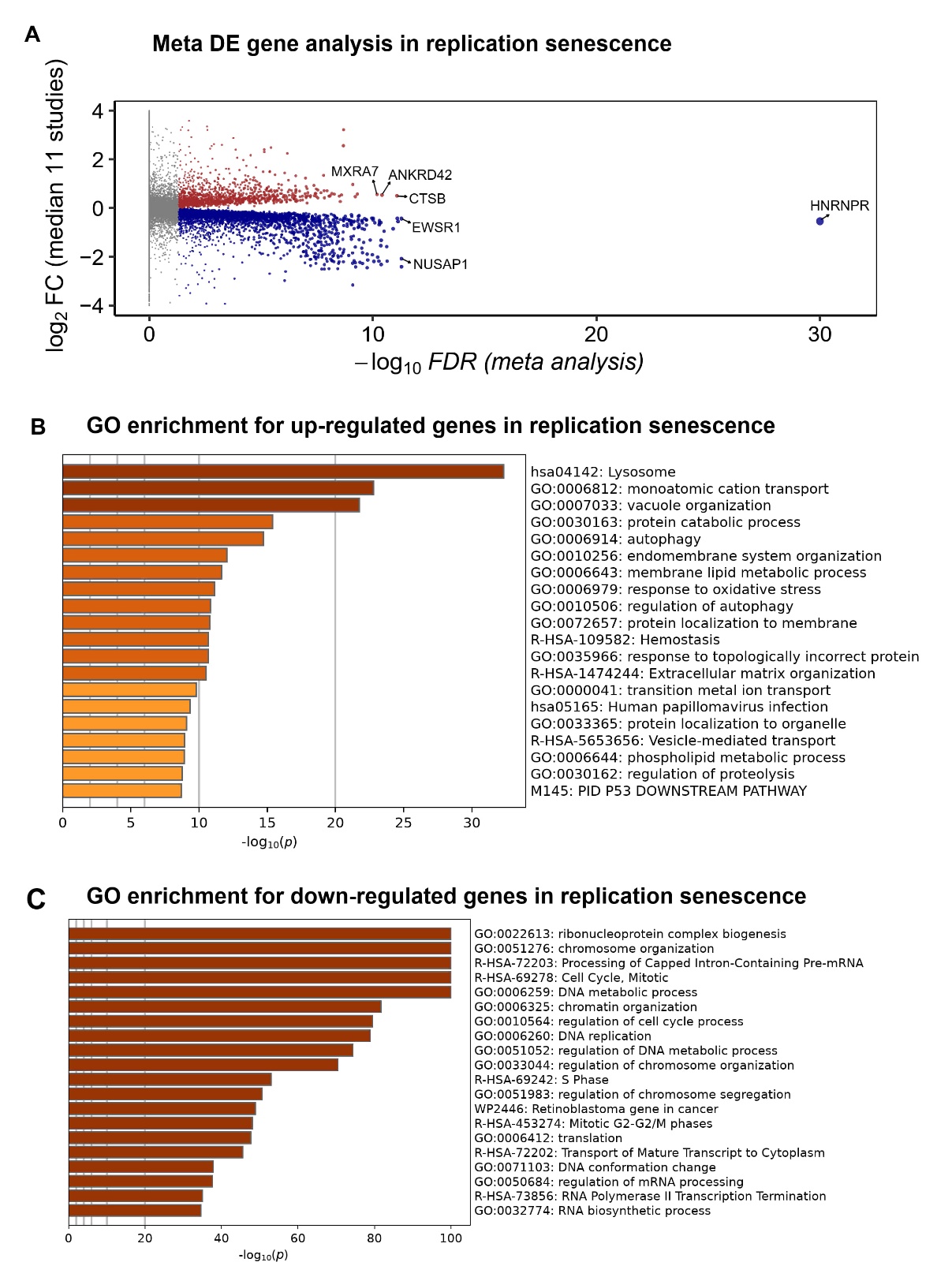


Figure S5. GO enrichment analysis of meta differential gene expression in replication senescence using Metascape.

(A) Volcano plot of meta differential gene expression in replication senescence. The number of significant up- and down-regulated genes are 952 and 2619, respectively (FDR < 0.01). (B) GO enrichment analysis of the up-regulated genes using one of the traditional gene set analysis method Metascape. (C) GO enrichment analysis of the down-regulated genes using one of the traditional gene set analysis method Metascape.


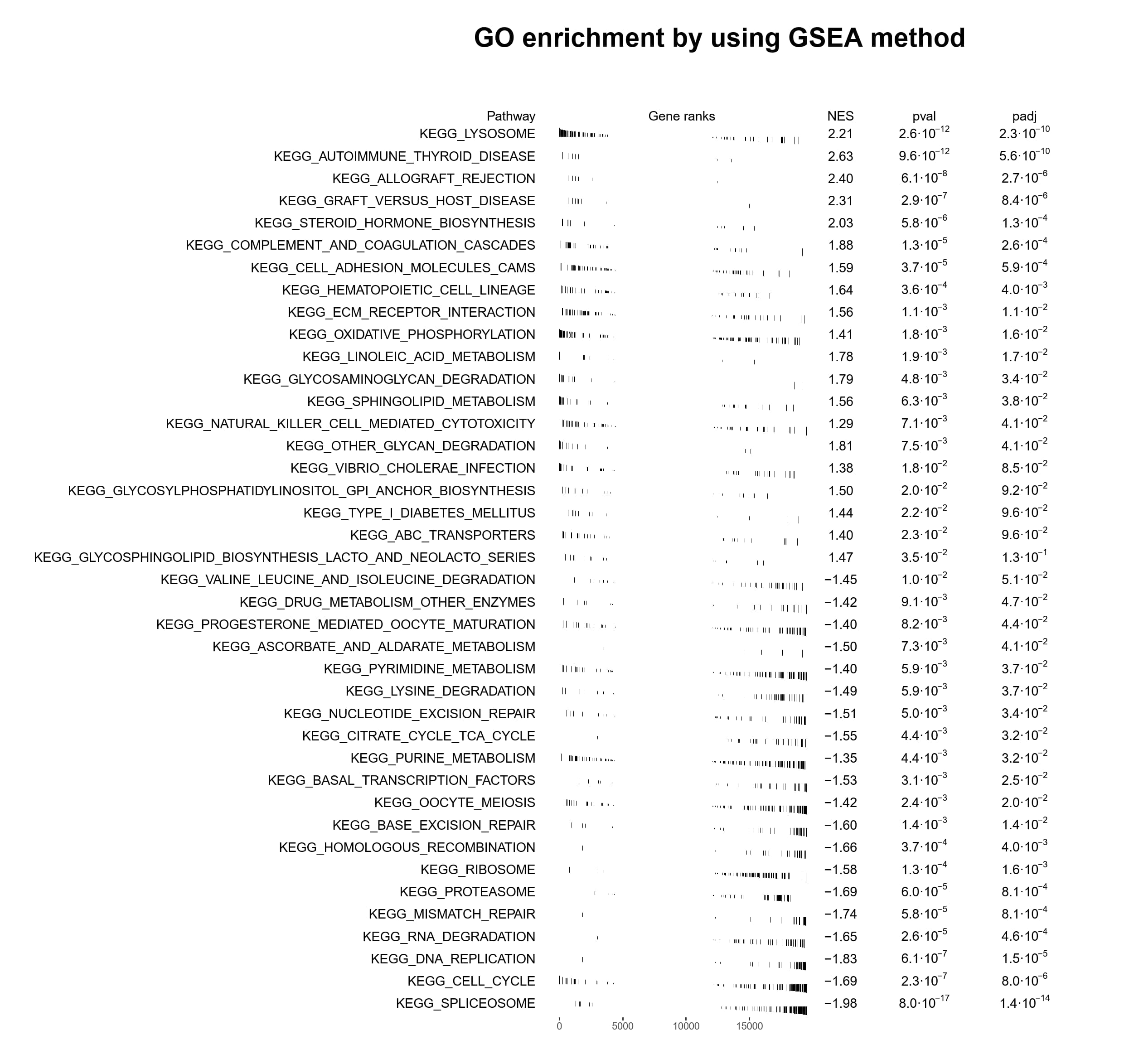


Figure S6. GO enrichment analysis of meta differential gene expression in replication senescence using GSEA method.


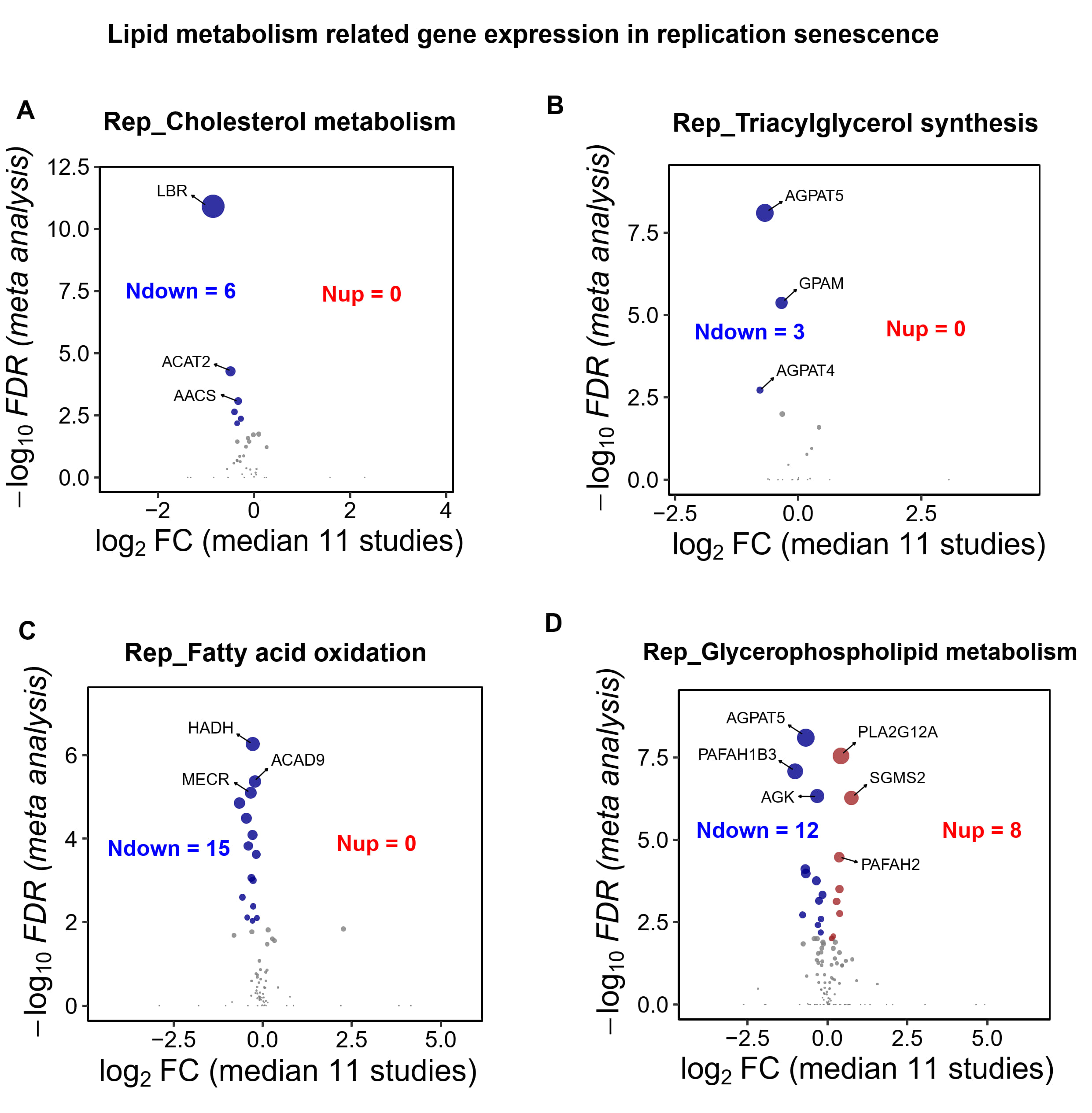


Figure S7. Volcano plot of lipid metabolism related genes in replication senescence.

(A) Cholesterol metabolism. (B) Triacylglycerol synthesis. (C) Fatty acid oxidation. (D) Glycerophospholipid metabolism.


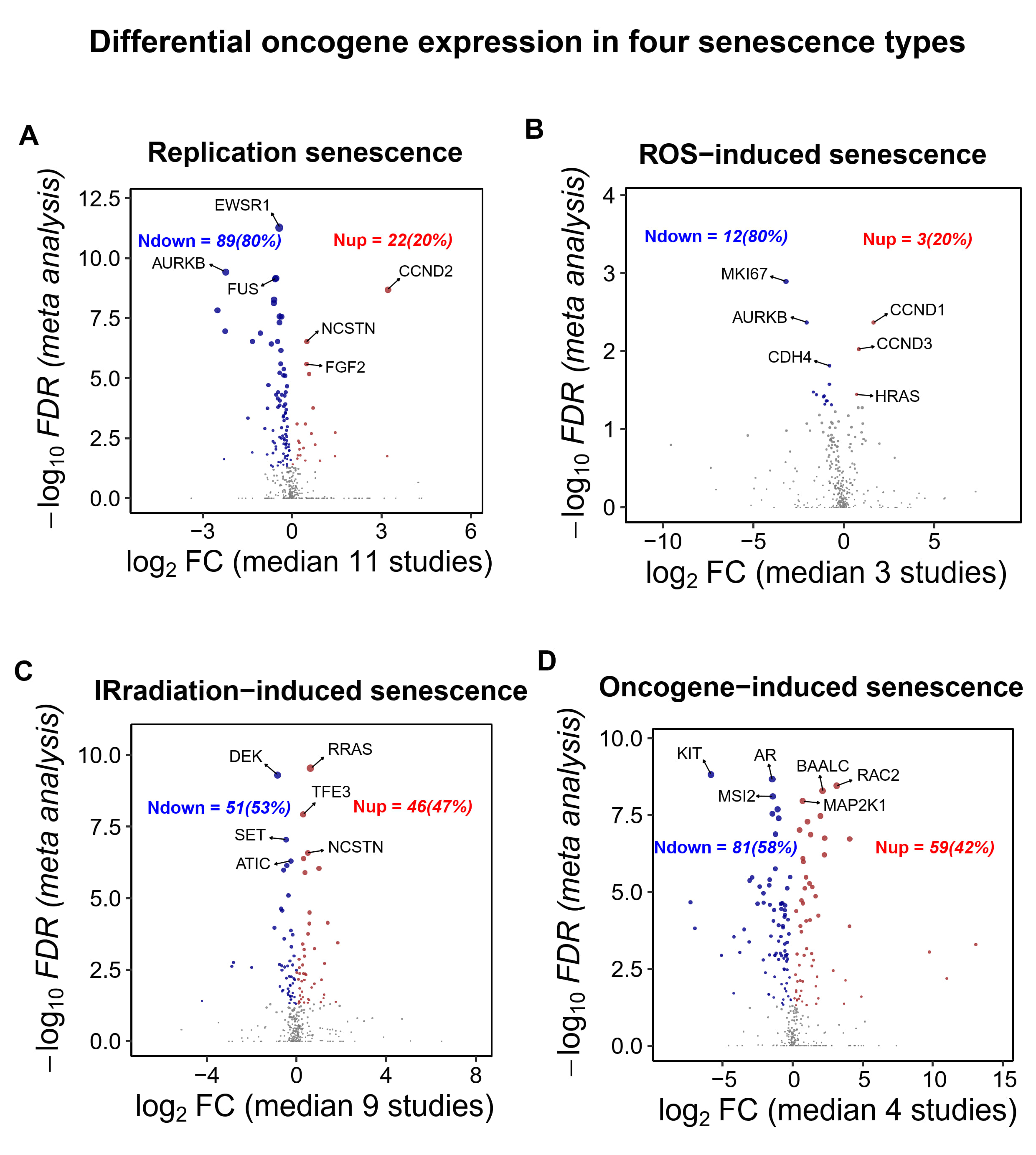


Figure S8. Volcano plot of the differential oncogene in different senescence types.

(A) Replication-induced senescence. Number of up and down regulated oncogenes are 22(20%), and 89(80%) respectively. (B) ROS-induced senescence. Number of up and down regulated oncogenes are 3(20%), and 12(80%) respectively. (C) IRradiation-induced senescence. Number of up and down regulated oncogenes are 46(47%), and 53(53%) respectively. (D) Oncogene-induced senescence. Number of up and down regulated oncogenes are 59(42%), and 81(58%) respectively. The list of oncogenes was downloaded from https://www.oncokb.org/cancer-genes, and filtered by oncogene only and overlapped with the identified genes in the study, the number of final filtered oncogenes is 349.
